# Graphene oxide scaffolds promote functional improvements mediated by scaffold-invading axons in thoracic transected rats

**DOI:** 10.1016/j.bioactmat.2024.12.031

**Published:** 2025-01-10

**Authors:** Marta Zaforas, Esther Benayas, Raquel Madroñero-Mariscal, Ana Domínguez-Bajo, Elena Fernández-López, Yasmina Hernández-Martín, Ankor González-Mayorga, Elena Alonso-Calviño, Eduardo R. Hernández, Elisa López-Dolado, Juliana M. Rosa, Juan Aguilar, María C. Serrano

**Affiliations:** a Laboratorio de Neurofisiología Experimental, Hospital Nacional de Parapléjicos, Finca La Peraleda s/n, 45071, Toledo, Spain; b Instituto de Ciencia de Materiales de Madrid (ICMM), Consejo Superior de Investigaciones Científicas (CSIC), Calle Sor Juana Inés de la Cruz 3, 28049, Madrid, Spain; c Laboratory of Interfaces for Neural Repair, Hospital Nacional de Parapléjicos, SESCAM, Finca La Peraleda s/n, 45071, Toledo, Spain; d Design and development of biomaterials for neural regeneration, HNP, Associated Unit to CSIC through ICMM, Finca La Peraleda s/n, 45071, Toledo, Spain; e Neuronal Circuits and Behaviour Group, Hospital Nacional de Parapléjicos, 45071, Toledo, Spain; f Instituto de Investigación Sanitaria de Castilla-La Mancha (IDISCAM), Spain; g Escuela de Doctorado UAM, Centro de Estudios de Posgrado, Universidad Autónoma de Madrid, Calle Francisco Tomás y Valiente 2, Ciudad Universitaria de Cantoblanco, Madrid, Spain

**Keywords:** Complete thoracic transection, Electrophysiological recording, Graphene oxide, Neural tissue engineering, Scaffold

## Abstract

Millions of patients and their caretakers live and deal with the devastating consequences of spinal cord injury (SCI) worldwide. Despite outstanding advances in the field to both understand and tackle these pathologies, a cure for SCI patients, with their peculiar characteristics, is still a mirage. One of the most promising therapeutic strategies to date for these patients involves the use of epidural electrical stimulation. In this context, electrically active materials such as graphene and its derivates become particularly interesting. Indeed, solid evidence of their capacity to closely interact with neural cells and networks is growing. Encouraged by previous findings in our laboratory on the exploration of 3D porous reduced graphene oxide (rGO) scaffolds in chronic cervical hemisected rats (C6), herein we report their neuro-reparative properties when chronically implanted in complete transected rats (T9-T10), in which no preserved contralateral neural networks can assist in any observed recovery. Electrophysiological recordings from brainstem regions show antidromic activation of a small population of neurons in response to electrical stimulation caudal to the injury. These neurons are located in the Gigantocellular nucleus of reticular formation and vestibular nuclei, both regions directly related to motor functions. Together with histological features at the lesion site, such as more abundant and larger blood vessels and more abundant, longer and more homogeneously distributed axons, our results corroborate that rGO scaffolds create a permissive environment that allows the invasion of functional axonic processes from neurons located in brainstem nuclei with motor function in a rat model of complete thoracic transection. Additionally, behavioral tests evidence that these scaffolds play an important role in whole-body mechanical stabilization (postural control) proved by the absence of scoliosis, a higher trunk stability and a larger cervico-thoraco-lumbar movement range in rGO-implanted rats.

## Highlights

• RGO scaffolds create a permissive environment that allow the invasion of functional axonic processes in a rat model of chronic complete thoracic transection.
• RGO scaffolds support more abundant and larger blood vessels and more abundant, longer and more homogeneously distributed axons at the injury site.
• Electrophysiological recordings from brainstem regions show antidromic activation of neurons in response to electrical stimulation caudal to the injury.
• These neurons are located in the Gigantocellular nucleus of reticular formation and vestibular nuclei, both regions directly related to motor functions.
• RGO scaffolds play an important role in whole-body mechanical stabilization (postural control).

## Graphical abstract

**Figure:**
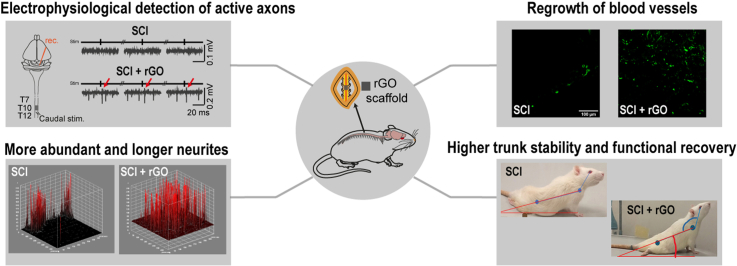

## 1 Introduction

First isolated in 2004 by Geim and Novoselov [1], the bidimensional nanomaterial graphene and its derivatives keep attracting much attention from scientists in a plethora of fields, including biomedical applications [2]. Impact is being particularly encouraging in the case of neural repair [3]. Indeed, the design of neural technologies has found promise on this type of nanomaterials. For example, Garrido and co-workers have developed microelectrodes based on nanoporous graphene thin-films of low impedance (∼25 kΩ) and high charge injection (3–5 mC cm^−2^). These microelectrodes allowed for high-precision and high-resolution neural interfacing *in vivo* [4], with no noticeable toxicity after chronic epidural or sciatic nerve implantation. When explored for neural tissue engineering, graphene-based materials (GBMs) have a proved capacity to create permissive substrates for neural growth both *in vitro* and *in vivo*. For instance, GBMs prompt neural differentiation from both neural and non-neural progenitor cells [5,6]. They also potentiate neurites sprouting and outgrowth [7], and stimulate electrical signaling in neural networks [8]. From a mechanistic point of view, Ballerini and co-workers provided outstanding insight into the ability of these nanomaterials to reshape neuronal synapse function [9], and to increase neuronal firing by tuning the distribution of extracellular ions at the membrane surface [10]. In the shape of 3D scaffolds, our group have pioneered the exploration of reduced graphene oxide (rGO) foams, only composed by rGO assembled flakes without further chemical or biological additives, for neural tissue engineering at the injured spinal cord. Promising findings included immunomodulatory effects [11] and the ingrowth of blood vessels and neurites within the lesion site [12] in chronic cervical hemisected rats. Efforts carried out by our group and others have also demonstrated that rGO prevents the extension of the lesion by reducing the perilesional damage [12], presents a beneficial adhesion to the spinal cord tissue [13], and does not induce local or systemic toxicity after subacute and chronic implantation [11].

Spinal cord injuries (SCIs) are devastating pathological conditions that radically transform the life of both patients and caretakers, often leading to life-threatening complications. Indeed, a total of 27 million people live with SCI worldwide, without significant variation in the age-standardized prevalence of the disease in the last 25 years [14]. Although an extensive effort is being done in the last decades by both clinicians and scientists to comprehend and address SCIs [3,15], none of the approaches under investigation has yet generated an effective therapy for these patients. Among the most promising advances in the field, epidural electrical stimulation seems at present to be closest to providing a cure for SCI patients [16,17]. Importantly, a certain cell population governing neural repair at the injured spinal cord has been recently identified [18] and the Tabulae Paralytica has been generated to dissect the molecular logic that governs the responses to injury within the spinal cord [19]. This priceless knowledge at the cellular and molecular levels in the context of SCI represents a breakthrough in the field that will undoubtedly benefit the design of a new generation of personalized treatments to address neural repair at the injured spinal cord. Other strategies based on cell therapy [3,20] and biomaterials other than GBMs [21,22] are also encouraging. In the case of cell therapy, the frequency of life-threatening adverse events following clinical trials in chronic SCI patients is very scarce [23]. Regarding the biomaterial of selection, a recent systematic review and network meta-analysis has revealed that natural scaffolds and synthetic scaffolds are equally effective in cell transplantation in SCI rats [24]. However, much work is still needed to bring cell therapy and biomaterials forward on the race to reach a SCI cure. Based on the promising findings generated by electrical stimulation approaches carried out by Courtine and others, and the beneficial capabilities of GBMs in the neural environment, the exploration of electrically active biomaterials such as GBMs continues to be particularly relevant for SCI repair.

In this work, we further test 3D rGO scaffolds in a rat model of complete thoracic transection, so, in contrast to previous work in a hemisection model, no contralateral neuronal and vascular collaterals could be responsible for any reparative features found. A thorough behavioral, electrophysiological and histological examination has been carried out. Rat motor behavior and any eventual functional improvements were evaluated by open field activity recording and two specifically defined body angle measurements. Electrophysiological studies included antidromic recordings after both rostral-to and caudal-to-lesion stimulations to physiologically identify the soma location where the axons invading the scaffolds originate. Histological examination focused on Masson's trichrome staining for collagen and cavities quantification and immunofluorescence labelling of selected neural, vascular and inflammatory components at the lesion site and its surroundings. Results were interpreted in an integrative manner and finally associated to data obtained from a cohort of comparable low thoracic SCI patients in an attempt to provide a translational meaning to our findings.

## 2 Material and Methods

### 2.1 Material

GO slurry was purchased from Graphenea, S.A. (Batch #C1250/GOB125/D; 4,6 wt% concentration, >95 % monolayer content). Neural cell culture media and supplements were acquired from Fisher Scientific. Chemical reagents were purchased from Sigma-Aldrich. All reagents were used as received unless otherwise indicated.

### 2.2 rGO scaffold preparation and physico-chemical characterization

3D rGO scaffolds were fabricated by a freeze-casting methodology as previously published [12]. Briefly, GO slurry was gently dispersed in distilled water (10 wt%) and pipetted into tap covers of 0.5 mL Eppendorf tubes (50 μL). Samples were then frozen at −80 °C overnight and subsequently freeze-dried for 24 h. The resulting cylindrical and porous monoliths were treated at 200 °C for 30 min. Prior to implantation, rGO scaffolds were sterilized under UV radiation (30 min) in a safety cabinet and hydrated in sterile cell culture grade water at 4 °C until complete immersion occurred (*ca.* 24 h). The physico-chemical characterization of these rGO scaffolds was carried out as previously described to confirm the equivalence of these scaffolds as compared to those previously explored in our laboratory [12]. For conductivity measurements, bigger rGO scaffolds were needed to meet the requirements of the equipment. The process of fabrication was similar to that described above, but in this case the taps of 2 mL Eppendorf tubes were used instead, resulting in monoliths of 70 mm in height x 40 mm in diameter. Measurements were carried out using a four-point probe (Ossila, UK). A total of 4 scaffolds were analyzed, performing 4 measures of 200 repetitions for each. The current range was set to a maximum of 200 μA and the maximum voltage to 5 V.

### 2.3 Animals

Experiments were performed in accordance with the European Union guidelines (Directive 2010/63/EU). All processes were approved by the Ethical Committee for Animal Research at the *Hospital Nacional de Parapléjicos* and the *Dirección General de Agricultura y Ganadería* of *Castilla-La Mancha* (reference numbers 21–2016, 20–2021, and 3–2023). A total of 32 male Wistar rats (3.1 ± 0.9 months old) were used (n = 5 for preliminary tests of the experimental approach, n = 7 died due to complications during surgery or post-surgery, n = 4 were processed as controls for histological studies, and n = 16 were used for electrophysiological and histological analysis). Wistar rats were submitted to a full transection of the spinal cord (T9-T10) and randomly assigned to two experimental groups: 1) full-transection without scaffold (n = 5; SCI), and 2) full-transection with rGO scaffold implantation at the injury site (n = 15; SCI + rGO). All animals were housed in standardized cages (2 per cage), with food and water *ad libitum* in a non-enriched environment and kept at 23 °C on a 12 h light/dark cycle.

### 2.4 SCI surgery and rGO scaffold implantation

Animals were anesthetized with pentobarbital (50 mg/kg *i.p.*) and administered xylazine (10 mg/kg *i.p.*) as a muscle relaxant and analgesic. The body temperature was kept constant (36.5 °C) using an automatically controlled heating pad (Cibertec SL, Madrid, Spain) and the eyes were protected with ophthalmic gel (Lubrithal, Dechra). Once rats were anesthetized, the skin of the surgical area was shaved, swabbed with ethanol 70 % and disinfected with Povidone iodine. A midline incision was made in the dorsal thoracic region over vertebral T8-T11 levels. Then, layers of muscles were carefully retracted keeping both the musculature and main blood vessels in the surgical area intact. Once vertebral bones were exposed, a laminectomy was performed at the thoracic level T9–T10, which allowed direct access to the spinal cord. After cutting the meninges for accessing the spinal cord, a complete transection (2 cm in length in the rostro-caudal axe) was carried out at this level of the cord using surgical microscissors and a curved needle and visually confirmed under the surgical microscope by the total separation of the spinal tissue borders. Transected animals were then randomly assigned to an injured-only (SCI) or an injured-implanted group (SCI + rGO). In implanted rats, a cylindrical and porous rGO scaffold (4 x 2 × 2 cm^3^) was carefully placed inside the injury site. The dimensions of the rGO scaffold (comparable to the size of the lesion) and its softness permitted its easy accommodation inside the empty space created in the transection, filling it adequately. A portion of autologous fat tissue was used to cover the complete spinal segment on top of the lesion site (with or without scaffold) in order to prevent the top muscular layers to invade the lesion. Dorsal muscular layers were smoothly approached and the skin closed using biodegradable sutures. Before the animals were awakened, a post-surgical treatment was applied using marbofloxacin (5 mg/kg s.c.), meloxicam (1 mg/kg s.c.), buprenorphine (0.05 mg/kg s.c., Indivior Europe Limited), and saline solution (B. Braun Medical, S.A.). From the first day after the surgery (and up to 5 days) animals received marbofloxacin, meloxicam and saline solution (same dose as previously described). Bladder voiding was practiced until normal voiding responses returned.

### 2.5 Surgery for electrophysiological recording sessions

Four months after SCI (range 3.6–4.6 months), acute antidromic electrophysiological recordings of bulbospinal neurons projecting to the spinal cord were performed. Distance between SCI and lambda cranial reference was 6.79 ± 0.62 cm (range 5.5–7.7 cm). For electrophysiological recordings, animals were first anesthetized with a mixture of isoflurane in oxygen (1.5–2 %, 1.2 L/min) applied by a mask (Medical Supplies & Services, Int. Ltd, UK), and the head was fixed in a stereotaxic frame with ear bars (SR-6R; Narishige Scientific Instruments, Tokyo, Japan). The body temperature was kept constant (36.5 °C) using an automatically controlled heating pad (Cibertec SL, Spain). The percentage of anesthetic was adjusted to induce and maintain a cortical state of slow wave activity (<1Hz) [25]. Cortical state was controlled by an electroencephalographic signal (EEG) obtained with a screw implanted over the primary somatosensory cortex [26]. In a first step, vertebral bones were exposed at the mid thoracic level and an extensive laminectomy of T7-T12 vertebras was carried out to allow a direct view of the injured spinal cord (T9-T10), as well as its respective rostral and caudal regions (T7-T8 and T11-12, respectively). In a second step, a craniotomy was performed over the stereotaxic coordinates of the reticular formation of the brainstem (anteroposterior, AP, from −1 to −4 mm and mediolateral, ML, from 0 to 3 mm using lambda as reference) [27], which allowed to access the brainstem for electrophysiological recordings using a tungsten electrode.

### 2.6 Spinal cord electrical stimulation

For the electrical stimulation of the spinal cord, two bipolar electrodes (parallel tips, 0.5 mm separated, 30G; B. Braun, Germany) were used. Using a micromanipulator, one electrode was placed in the rostral segment (T7-T8) relative to the lesion and/or scaffold location (T9-T10), and the other in the caudal (T11-T12) segment. The rostral and caudal distance from the electrode to the injury location was 0.7 ± 0.2 cm (range 0.3–1 cm) and 0.5 ± 0.2 cm (range 0.2–0.7 cm), respectively. Electrode placement was intended to activate spinal cord tracts ipsilaterally to the recording electrode, so one pole was placed in the midline and the other in the most lateral part of the spinal cord. Square electrical pulses of varied duration, frequency and intensity (0.1–1 ms, 0.5–500 Hz, 0.1–5 mA) were applied using a digital stimulator (Master 8, A.M.P.I.) connected to an isolation unit (ISO-Flex, A.M.P.I.).

### 2.7 Electrophysiological recordings and analyses

Individual neurons were recorded with a high impedance (4–5 MΩ) tungsten electrode (TM31C40KT and TM31A50KT, World Precision Instruments Inc., Sarasota, FL, USA), lowered into the brainstem using a stereotaxic hydraulic micromanipulator (SM-15 and MO-10; Narishige Scientific Instruments, Tokyo, Japan). Raw signals were filtered online (0.5 Hz–3 kHz) and amplified ( × 500) using a modular system of a preamplifier, filter and amplifier (Neurolog System, Digitimer Ltd., UK). Recordings were digitalized online for direct visualization using an A/D converter (1401 CED Cambridge Electronic Designs, UK) controlled by the Spike2 software (Spike2 v7, RRID:SCR_000903, Cambridge Electronic Design CED, UK). All the data were stored on an external hard disk for posterior off-line analysis. In 1 out of 16 experiments, no recording of antidromic activity were obtained in any neuron, so recordings from 15 animals were analyzed.

**Fig. 1:**
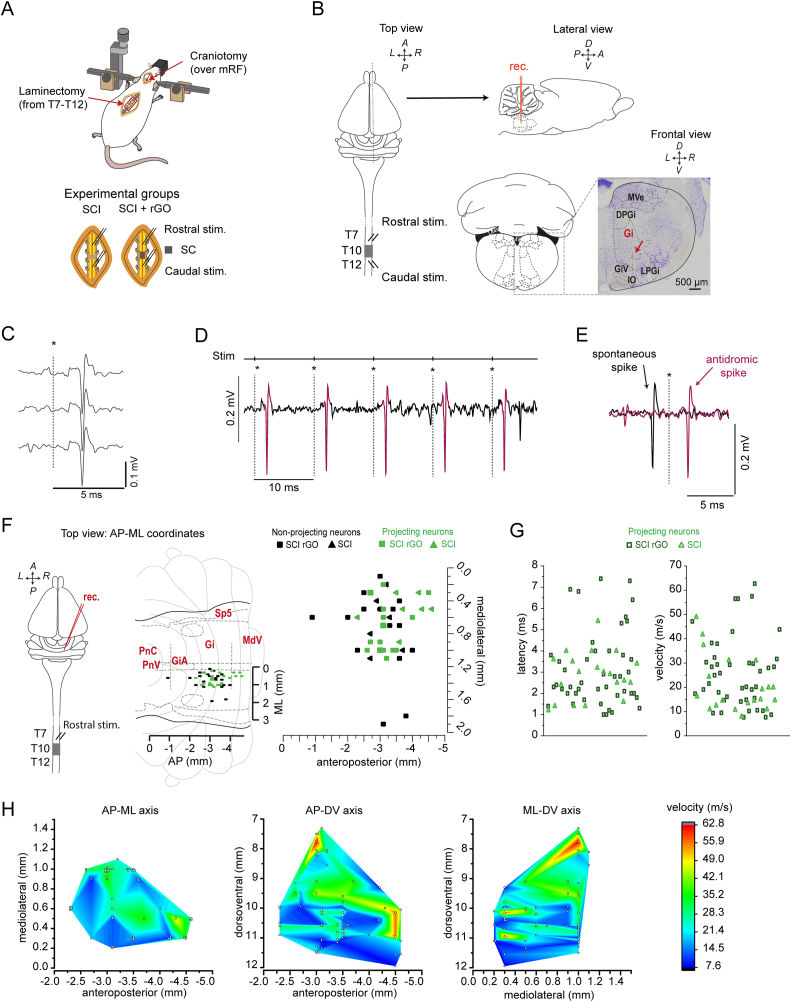
**Experimental approach and identification of spinal cord projecting neurons. (A)** Experimental approach for electrophysiological recordings of spinal cord-projecting neurons and stimulation of their axons at spinal cord level in adult Wistar rats with a complete spinal cord injury at mid thoracic level (T9-T10). Animals were randomly assigned to two experimental groups the day of SCI surgery: only-injured (SCI) and implanted with a graphene scaffold in the site of injury (SCI + rGO). **(B)** Three different spatial schematic views of experimental approach (top, lateral and frontal) with the histological demonstration of an electrode insertion, performed by a Nissl staining of a 50 μm coronal slice at a spatial coordinate of AP -3 with lambda as a reference [27]. **(C**–**E)** Antidromic characterization of spinal-cord projecting neurons (see Methods): constant latency and amplitude (C), follows high frequency stimulation (>100 Hz, D) and finally, neuron passes the collision test (E), which means that if a spontaneous orthodromic action potential occurs, it blocks the generation of antidromic spike. **(F)** Left, top view of scheme in B showing rostral stimulation. Middle and right: distribution plot of the 65 antidromic neurons (green) that were found in 60 electrode insertions made in 16 animals (square: SCI + rGO group, triangle: SCI group). These neurons are plotted as a function of AP and ML spatial coordinates. **(G)** Physiological properties of antidromic neurons identified by rostral stimulation in the brainstem: latency (ms) and conduction velocity (m/s). **(H)** Heat maps showing velocity of antidromic neurons as function of three spatial coordinates (AP-ML, AP-DV and ML-DV from left to right). Note that DV axis differentiates two nuclei with high conduction velocity in the spatial coordinates of vestibular and Gigantocellular nuclei, both in medulla. A: anterior; AP: anteroposterior; D: dorsal; DV: dorsoventral; L: left; ML: mediolateral; P: posterior; R: right; V: ventral.

After placement of the stimulation electrode at the caudal and rostral segments of the SCI (location indicated above, Fig. 1A and B), the recording electrode was initially lowered at the stereotaxic coordinates of the Gigantocellular nucleus (Gi): AP -1.3 to −4.3 mm; ML 0–1.8 mm (relative to lambda/interaural cranial reference) [27]. Then, square pulses (see above) were applied to the rostral segment of the SCI to identify those single neurons showing antidromic responses (*i.e.* consistent action potentials) to the stimulation (see Results for details of single neuron antidromic activation). After the antidromically identification of the single neurons, a protocol of increased stimulation frequencies (1–500 Hz) at a fixed intensity was applied. The intensity of the stimulation protocol for the rostral segment was fixed for each neuron at a value 50 % above the threshold for activation (Fig. 1C-E). If no antidromic responses were observed, the recording electrode was removed and lowered at different structures of the brainstem following coordinates in three stereotaxic axes: anteroposterior, dorsoventral and medio-lateral (AP, DV and ML, respectively), in order to obtain a map of antidromic responding neurons (Fig. 1F–1H).

Once a neuron was isolated and identified as antidromically activated by stimulation of the rostral location, the stimulation was applied in the caudal location to the lesion (with/without rGO) to determine if the same neuron could be activated, therefore confirming that the same axon activated by rostral stimulation was invading the scaffold. For the stimulation of the caudal segment, a standardized protocol was first applied with a high intensity and low frequency stimulation: 5 mA, 0.5–2 Hz and a pulse duration of 0.5 ms. If an obvious response of the neuron was found, as for the rostral segment, a stimulation protocol of increased frequencies was applied, to confirm the unequivocal antidromic activation of the same axon.

To identify antidromic action potentials in response to electrical stimulation, raw signals were filtered with a band pass of 500–5000 Hz. Then, a voltage threshold value was determined from the mean + 3 SD of the background signal recorded to isolate the action potential from such single neuron. Visual inspection of the action potential waveform was then performed trial by trial to verify the response. Then, the efficiency of antidromic responses was calculated from a peristimulus time histogram (PSTH) across stimuli (1 ms bin), which is represented as the percentage of responses from the total number of stimuli. The same process was applied to study neuronal responses to electrical stimulation caudal to the lesion (with/without rGO). Units were considered as respondent to caudal stimulation when a single bin overcame the threshold based on the mean + SD obtained from the background, *i.e.* activity previous to the stimulation.

### 2.8 Behavioral tests

Behavioral tests were used to detect changes in both infralesional motor deficits and whole-body motor compensations after 4 months of implantation. The Basso, Beattie and Bresnahan (BBB) score, as gold standard for evaluating rat locomotion after thoracic spinal cord transections, and the open field test, a classic method to evaluate exploratory behavior in rodents, were used. As grooming is a very important activity in rodents and can involve different body patterns implying both the use of forepaws and trunk strength, a test quantifying the grooming activity of the rats was also applied, as previously described [12]. Additionally, two angular measures of body alignment were defined to determine whether the mechanical stabilization driven by the scaffold had an impact on body alignment and, consequently, on motor behavior.

#### 2.8.1 The BBB open field locomotion score

The BBB locomotion score was developed to assess the gait of thoracic spinal cord injured rats [28,29]. This scale goes from a score of 0, which indicates no spontaneous movement, to a score of 21 that indicates normal locomotion. Plantar gait with full weight bearing and four limb coordination is achieved when an animal reaches ≥14 points. Prior to scoring sessions, rats were conditioned to walk in a circular open field consisting of an 80 × 130 cm^2^ table limited by clear Plexiglass walls with 30 cm in height and a non-slippery floor. Immediately prior to electrophysiological recordings, 4-min test sessions were carried out using the BBB scale by two independent observers.

#### 2.8.2 The open field test

The open field test was performed by recording the spontaneous activity of rats located on the same round table used for BBB tests in a quiet room, as previously described [30]. For each session and rat, the video recordings lasted 10 min. Rat behavior was recorded by using two digital cameras strategically located around the table to cover 360°. The acquired videos were used to quantify the relative percentage of active behavior, locomotion and grooming for each tested animal.

#### 2.8.3 Grooming activity

Spontaneous grooming activity was quantified from the videos recorded during the open field tests. Stereotyped grooming sequences included (1) forepaw licking and face washing, (2) forepaw face grooming, (3) repetitive body licking, and (4) hind paw scratching, as described elsewhere [31], and previously performed in our laboratory [12]. A slow-motion mode was used to score the movements of the different forepaws according to the maximum contact made at the initiation of each part of the grooming sequence.

#### 2.8.4 Angular alignment measures

From all video recordings, a selection of frames with a complete lateral view of the rat (orthogonal to the recording camera) was done (N ≥ 3 per animal). Prior to recordings, the points corresponding to the shoulder and hip joints on both sides were located by palpation and used as body references, together with the eye representing the head, for the definition of two different angular alignment measures. On the one hand, the intersection of the line joining the eye to the shoulder and the shoulder to the hip defines the *cervical-thoracic-lumbar angle*. The amplitude of this angle primarily depends on cranio-cervical, forepaw and upper body strength, ranging from the position in which the rat's neck is in maximum flexion (*i.e.,* the snout closest to the ground) to the position of maximum extension with the head raised and the snout pointing upwards. The value of this angle is representative of the motor function of the forepaws and upper half of the trunk, exactly the body area above the lesion in the complete thoracic SCI model chosen for the present study. On the other hand, the intersection of the line joining the shoulder and the hip with the horizontal line defined by the ground represents the *trunk alignment angle*. The value of this angle is representative of the motor function of the hindpaws and the pelvis, which is the body area below the lesion in the injury model of choice.

### 2.9 Histological studies

At the end of the electrophysiological experiments, rats received an overdose of pentobarbital (50 mg/kg, intraperitoneal) and transcardially perfused with 0.1 % heparin in phosphate buffer saline (PBS) solution followed by paraformaldehyde (4 % in PBS). All perfused brain and spinal cord samples were placed in paraformaldehyde 4 % at 4 °C overnight and then 3 days in sucrose (30 % in PBS) at 4 °C for cryo-protection. Tissue pieces were mounted on plastic containers, quick-frozen in either Tissue Freezing Medium (brain; Leica) or Optimal Cutting Temperature compound (spinal cord; Tissue Tek). On the one hand, brain tissue corresponding to the brainstem was processed to verify the spatial location of the recording electrodes in the three brain axes (AP, ML and DV). Brainstem tissue was cut in coronal sections of 50 μm by using a freezing microtome (Microm HM 450, Microm International GmbH, Germany) from AP -1 to −4 mm (relative to lambda/interaural cranial reference) [27]. Following washing in 0.1 M phosphate saline, sections were mounted on gelatin slides, air-dried, processed for tionine (Nissl) staining (Sigma-Aldrich), dehydrated in xylene and coverslipped with DePeX (SEVA, Germany). On the other hand, spinal cord tissue was cut in horizontal sections of 10 μm by using a Leica CM1900 cryostat with an angle of 10°. In the particular case of spinal cords, the entire spinal fragment was cut in sagittal sections from right to left.

Spinal cords were examined after Masson's trichrome for quantification of cavities and visualization of collagen. In all cases (brain and spinal cord), panoramic (low magnification) and zoom-in images were collected by using an Olympus BX61 microscope. In order to quantify the level of collagen compactness a clustering analysis of Masson's trichrome images was first done, classifying each pixel according to its dominant colour. The clustering analysis identified a pre-defined number of clusters, *i.e.* colours, which in this case was defined at 16, as the most representative in the image. The clustering analysis was performed using the K-Means algorithm [32] as implemented in the Scikit-Learn python library [33]. Each pixel was then assigned to the colour closest to its own. In this way the image could be segmented into pixels that corresponded to the substrate or background, and pixels that corresponded to the sample; the latter could be further subdivided into red-purple colours, corresponding to sample with no collagen, and blue colours, where collagen was present. Knowing the area per pixel, the areas of each region in the sample were quantified. As a measure of the intensity of blue, the inverse of the corresponding grey-scale colour of the blue pixels was taken, since in grey-scale the highest value (255) corresponds to white. To quantify the compactness of a given blue pixel, the summed intensities of its neighbouring pixels (intensities of non-blue pixels taken as zero) were accumulated to its own value. Thus, areas with a dense accumulation of collagen were represented by pixel areas with high values of summed intensity. The Python script used to perform the collagen compactness analysis described in the text can be freely downloaded from the following link: https://github.com/errhernandez/compactness.

### 2.10 Immunofluorescence studies

Spinal cord samples were examined for the presence of the following markers: (1) microtubule-associated protein 2 (MAP-2; M1406, 1:500, Merck) and (2) βIII-tubulin for somas and neurites in neurons (T2200, 1:500, Merck), (3) vimentin for non-neuronal cells including glial and connective tissue cells (SAB4300676, 1:500; Merck), (4) glial fibrillary acidic protein (GFAP) for astrocytes (G3893, 1:400, Merck), (5) ED1 for macrophages (MAB1435, 1:100, Chemicon), (6) RECA-1 for endothelial cells in blood vessels (MCA970R, 1:250, Bio-Rad), (7) vesicular glutamate transporter 2 (VGLUT2) for excitatory glutamatergic fibers (135–403, 1:500, Synaptic Systems), (8) SOX-10 for Schwann cells, (9) GAP-43 for neuronal growth cones, (10) synaptophysin for synapses detection, and (11) MAG (myelin-associated protein) and (12) MBP (myelin basic protein) for myelin labelling. Appropriate secondary antibodies were selected accordingly. In all cases, cell nuclei were visualized by labeling with Hoechst (1 mg mL^−1^). Fluorescence images were collected by using a Leica resonant SP5 microscope. Capture conditions were fixed by using sections from the three experimental groups incubated with the secondary antibodies without primary ones. All images were thereafter captured under these conditions. All fluorescence images were automatically quantified by using a customized macro in Fiji software as the number of pixels (μm^2^) positively stained for each particular fluorescence marker after the definition of the corresponding threshold of positive labeling. Control spinal cords served as reference values to define appropriate threshold values for each marker. At least three non-overlapping images per animal were acquired in each position of interest (N ≥ 9 per fluorescence marker, study region and group from at least 4 different animals per group). Areas under study were: perilesional areas at 1–2 mm from the lesion site – PL12-, caudal interface of the lesion – CIF-, rostral interface of the lesion – RIF-, and lesion site (with or without rGO scaffold). Bright field images were also acquired to define scaffold location. Quantification of number, diameter and length of blood vessels (RECA-1^+^) and number, rostro-caudal individual length and total length per image of neurites (βIII-tubulin^+^) was carried out. Finally, a procedure previously described for the calculation of the distributional homogeneity index (DHI) in hyperspectral images of solid pharmaceutical dosage forms was adapted for assessing the homogeneity of the colonization of neurites at the lesion site [34].

### 2.11 Data analysis from patients’ series

A cohort of 101 patients with traumatic chronic SCI recruited for ambispective follow-up of developing complications in the *Hospital Nacional de Parapléjicos* was used. From those, only 20 patients with a motor complete AIS A low thoracic (T8 – T12) SCI were selected, as these lesions are the only human lesions comparable in level and severity to the thoracic transection of the rats used in the present work. In these patients, motor and functional behavior was assessed and the eventual functional impact of either the presence or absence of spasticity examined. All subjects gave their signed informed consent for their data to be used, which was authorized by the Patients’ Institutional Review Board of the *Hospital Nacional de Parapléjicos* (September 10, 2015). The study was performed under the fundamental ethical principles of autonomy, beneficence, non-maleficence, and distributive justice and by the statements of good clinical practice, the tenets of the most recent Helsinki Declaration (2013), and the Oviedo Convention (1997). Data were gathered following current data protection laws including Regulation (EU) 2016/679 and Organic Law March 2018 of 5 December, which protects personal data. Medical information was collected in a routine clinical examination in the Physical Medicine and Rehabilitation Department of the *Hospital Nacional de Parapléjicos*.

The inclusion criteria were: (1) being ≥18 years-old, and (2) history of low thoracic (T8 – T12) motor complete AIS A SCI for more than 1 year. A certified clinician in SCI medicine evaluated the subjects’ injuries according to the International Standards for Neurologic Classification of Spinal Cord Injury [35]. Conversely, exclusion criteria were as follows: (1) a coincident infection with notable severity, such as urinary tract infection or a respiratory infection, evidenced with a positive culture in the last 3 months; (2) chronic viral or bacterial infection; (3) clinical diagnosis of an autoimmune disease; (4) serious cardiovascular disease; (5) hematopoietic, renal, lung, or hepatic complications; (6) an endocrine or metabolic disorder, (*i.e.,* type 1/2 diabetes mellitus); (7) previous history of cancer; (8) pressure ulcers in the last year; (9) administration of immunomodulatory drugs such as steroids in the last 3 months; (10) suffering from immunodeficiency or malnutrition; (11) being in a pregnancy or lactation period; and (12) having undergone diagnosis of any psychiatric disorder.

The following variables were studied in the selected patients: 1) demographic data such as gender, age, and time evolution of the SCI; 2) the presence/absence of infralesional spasticity, measured according to the Modified Ashworth scale [36]; 3) the residual locomotor capacity measured using the LEMS score [35] and WISCI II scale [37]; and 4) the degree of functional independence measured using the SCIM III scale, including both the total score and the scores of two of its subscales, self-care and mobility [38].

### 2.12 Statistics

All values (both preclinical and clinical) were expressed as the mean ± standard deviation of at least three different individuals per group (N ≥ 3). Electrophysiological data was graphed as scatter plots indicating all of the individual neurons recorded in the experiments. The rest of data (non-electrophysiological) was graphed in violin plots, which depict distributions of numeric data using density curves. The width of each curve corresponds with the approximate frequency of data points in each region. Single data points were overlapped with their correspondent violin plots. Statistical analysis was performed by using the Statistical Package for the Social Sciences (SPSS, version 29.0.0.0). Comparisons among groups were done by one-way analysis of variance (ANOVA) and either *post-hoc* Scheffé or Games-Howell tests (homogeneous *vs.* heterogeneous variances, respectively, as dictated by Levene's test). A T-test was used for comparisons between two independent groups with normal distributions. Alternatively, the Mann-Whitney rank sum test and two-sample Kolmogorov-Smirnov test were used if the normality test failed. For the analysis of dichotomous variables, χ^2^ test was applied. In all cases, the significance levels were defined as p < 0.05 (∗), p < 0.01 (∗∗) and p < 0.005 (∗∗∗).

## 3 Results

Previous work by our group demonstrated that rGO scaffolds, without any specific chemical or biological functionalization, promoted pro-regenerative features in chronic cervical hemisected rats (C6 level) [12]. Motivated by these encouraging findings, we herein explored the response to these same scaffolds (some details of their physic-chemical characterization are shown in Fig. S1) when implanted in a more challenging scenario, a spinal complete transection at the thoracic level (T9-T10), in which no preserved contralateral neural networks could assist any repair found. The time point of selection was a chronic lesion (4 months) as it was the one with the larger quantity of neuro-reparative features found in the cervical segment as described in previous work [11,12].

### 3.1 In vivo ephys recordings: rGO scaffolds promote electrical activation below the injury level

**Fig. 2:**
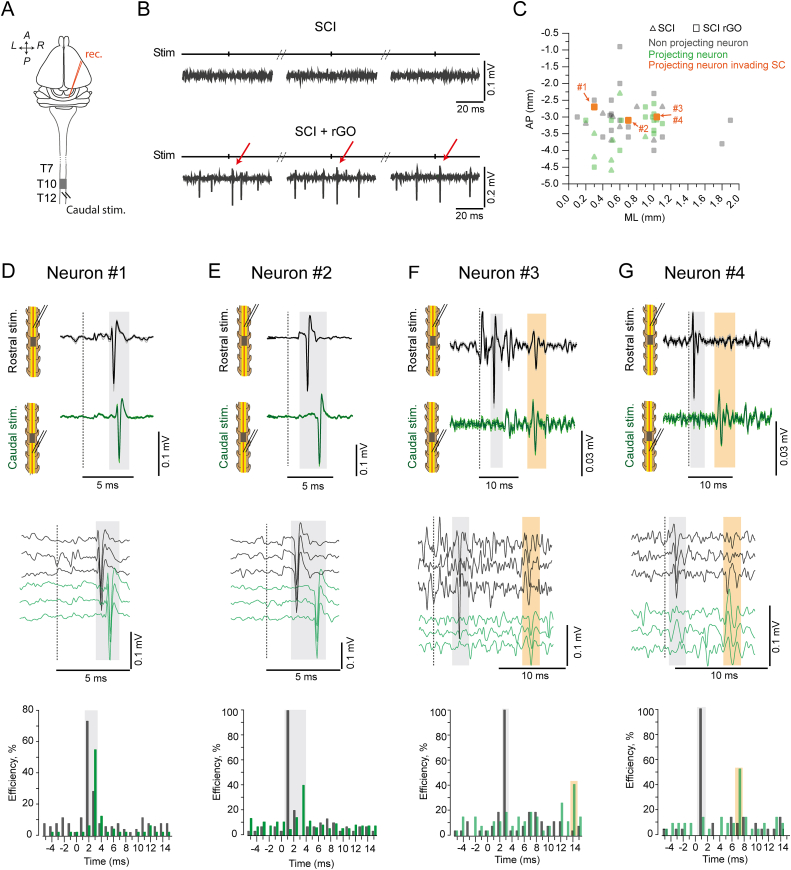
**Caudal activation of projecting neurons axons invading scaffold. (A)** Scheme showing experimental approach for recording and stimulation for caudal activation of projecting neurons. Stim.: stimulation, rec.: recording, A: anterior, P: posterior, L: left, R: right, T7-T12: thoracic vertebrae. **(B)** Three single trials of caudal stimuli (Stim) at low frequency (1–2 Hz) for an animal of SCI group (top) and one of SCI + rGO group (bottom). Traces show examples of filtered signals (band-pass 0.5–5 kHz). Red arrows indicate antidromic caudal activation of a neuron invading scaffold from the SCI + rGO group (neuron #2). **(C)** Distribution plot of AP-ML coordinates for electrode insertion (n = 60) where no projecting neuron was found (grey, n = 33) and those where projecting neurons were recorded (green, n = 27), with locations for projecting neurons that invaded scaffold highlighted in orange (n = 4). Numbered neurons invading scaffold are named as Neuron #1 to #4. **(D, E, F, G)** Characterization of Neuron #1 (D), #2 (E), #3 (F) and #4 (G). Spatial coordinates of Neurons #1-#4: AP -3, −2.7, −3 and −3 mm; ML 0.7, 0.3, 1 and 1 mm; DV 9.5, 10.6, 8.2 and 8 mm, respectively (see panel C). Dotted vertical line indicates stimulus timestamp and shaded black squares highlight antidromic neurons response while shaded orange squares indicate synaptic response in Neuron #3 (F) and #4 (G). Top: Response average (±SEM, lighter color) of 15–37 stimuli during low frequency (0.5–5 Hz) stimulation of rostral (black, top) and caudal (green, bottom) segments of spinal cord. Middle: Three single trials of filtered signal with neuron response for rostral (black) and caudal (green) stimulation. Bottom: Peristimulus histogram (PSTH) with 1 ms bin showing neuron efficiency (%), calculated as count of spikes/number of stimuli. PSTH was built using 30–50 stimuli and values for each bin were normalized to the total number of stimuli (%) in order to compare between neurons.

With the aim of corroborating the existence of spared neurons invading the scaffold at the thoracic level and proving their electrical activation, the first step in this study was to perform antidromic electrophysiological recordings from these rats both in the absence and presence of rGO implants (SCI and SCI + rGO, respectively; Fig. 1A and B). Electrical stimulation of the spinal cord segment rostral to T10 allowed us to identify neurons in the brainstem projecting to the spinal cord showing antidromic activation/responses. A recorded neuron was classified as respondent to antidromic stimulation (in rostral location) when it followed at least two of the three following criteria [39,40]: 1) constant latency and amplitude of action potentials (Fig. 1C); 2) reliable responses to high frequency stimulation (50–200 Hz, Fig. 1D); and 3) a positive collision test between antidromic and spontaneous orthodromic action potentials (Fig. 1E). A total of 60 recording locations were obtained from a total of 16 rats at different anteroposterior and mediolateral coordinates across the brainstem (Fig. 1F). From these rats, 65 antidromically activated neurons were found in 27 recording locations: 45 neurons from SCI + rGO animals (n = 11; 4.1 ± 2.7 neurons/animal) and 20 neurons from SCI animals (N = 4; 5.0 ± 3.7 neurons/animal). From one of the injured animals without rGO (SCI group), no neurons could be recorded. The pool of antidromically activated neurons by rostral stimulation showed a wide range of conduction velocities (7.6–62.7 m/s) and response latencies (1.0–7.4 ms), with no statistical differences between both groups (two-sample Kolmogorov-Smirnov test, p = 0.185 and p = 0.352 for velocity and latency, respectively) (Fig. 1G). Recorded neurons showing the highest conduction velocity were observed within the Gigantocellular (Gi) and vestibular nuclei in both groups, as shown in the heat map representations (Fig. 1H).

Once a neuron responded to rostral stimulation, the next step was to apply the stimulation to the caudal segment of the lesion/rGO to determine if the same axon was invading that region and was physiologically functional (Fig. 2A). No brainstem responses were observed when caudal stimulation was applied in any of the SCI animals, confirming the complete transection of the spinal cord (Fig. 2B and C). However, 27.3 % (3 out of 11 rats) of SCI + rGO animals exhibited at least 1 activated neuron in response to the stimulation of the caudal segment to the complete transected spinal cord (Fig. 2B and C). From these 3 animals, a total of 2 neurons exhibited the physiological parameters to be considered antidromically activated (Neuron #1 and #2) and the other two were considered as synaptically activated neurons (Fig. 2D–G and Fig. S2). Neuron #1 (AP -2.7 mm, ML 0.3 mm, DV 9.5 mm) and neuron #2 (AP -3.1 mm, ML 0.7 mm, DV 10.6 mm) were recorded from rats X21 and X23, respectively, and their stereotaxic coordinates were consistent with the Gi nucleus. These neurons exhibited slightly different properties to rostral and caudal stimulations (Fig. 2D and E). First, neuron #1 showed a latency of 3 ms and a conduction velocity to rostral stimulation of 19.1 m/s, with a 100 % of response efficiency when activated by low and high frequencies (>100 Hz, Figs. 2D and S2A). The collision test was also observed between the spontaneous orthodromic action potential and the antidromic activation (Fig. S2A). When neuron #1 was activated by caudal stimulation, it showed responses with a latency of 3.8 ms and a conduction velocity of 18 m/s, with a response efficiency of 55 % in response to low frequency stimulation (<5 Hz) (Fig. 2D bottom). Differently, neuron #2 - recorded from a different animal-showed a latency of 2 ms and a conduction velocity to rostral stimulation of 28.5 m/s, was activated at high frequencies (>100 Hz) with a 100 % of response efficiency and the antidromic activation was blocked by orthodromic action potential, confirming the collision test (Figs. 2E and S2B). The same neuron #2 activated by caudal stimulation showed 1.5 ms of delay with respect to rostral stimulation, which produced a slower conduction velocity (20.3 m/s) and a response efficiency of 47 % when the stimulation was applied at low frequencies (<5Hz).

Neurons #3 and #4 were found in the same experiment performed in rat X6 at the stereotaxic coordinates of AP -3 mm, ML 1 mm and DV 8.2 and 8 mm, respectively, corresponding to the vestibular nuclei containing neurons with direct axonal projections to the spinal cord [27]. In the case of neuron #3, the rostral stimulation produced clear antidromic action potentials (100 % efficiency at low and high frequencies) at a latency of 3.8 ms and a conduction velocity of 17.4 m/s. When stimulation was caudally applied, no distinguished antidromic responses were observed. However, we found two units activated only by low frequency showing long latencies (7.7 ms and 14.5 ms, respectively, Figs. 2F and S2) and low efficiency (19 % and 41 %, respectively) that failed the collision test. This indicates that the activity from the recorded neuron, rather than a direct antidromic activation, was due to a synaptic input from neighbor neurons in the same nucleus, whose axons were inside the scaffold and antidromically activated. These two units were also visually observed following rostral stimulation but with variable amplitude and lower efficiency compared to the antidromic response (Fig. S2C). Neuron #4 (DV 8 mm in the same animal; Figs. 2G and S2) showed similar responses to those obtained in neuron #3. Rostral stimulation of the injured spinal cord induced a clear antidromic response with 100 % of efficiency showing a short latency (1.4 ms) and a conduction velocity of 47.1 m/s. Similarly, caudal stimulation induced a consistent response showing a low efficiency (52.4 %) and a long latency (7.8 ms). The activity from these two neurons were characterized by a variable response amplitude of small local field potentials, indicating that the antidromic stimulation of different axons located inside the scaffold are synaptically activating a small cluster of neurons by its axonal collaterals within the same brain structure (*i.e.* vestibular nuclei).

### 3.2 Histological examination

**Fig. 3:**
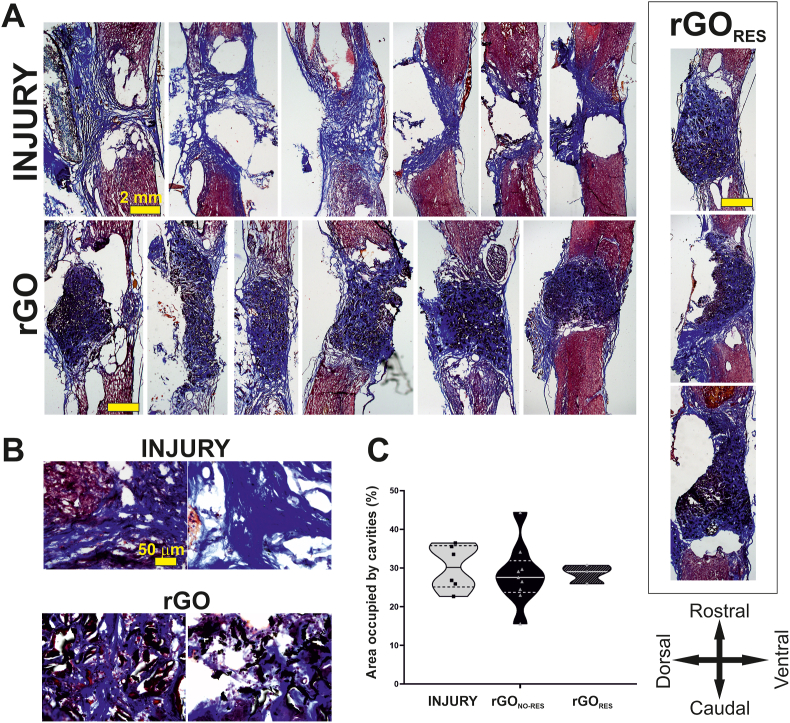
**Histological examination of spinal cord tissue from transected rats receiving rGO scaffolds. (A)** Representative Masson trichrome staining images of spinal cords after 4 months of implantation for the different experimental groups. Each image represents the spinal cord of a different rat within each experimental group. Scale bars: 2 mm. **(B)** Details of the lesion site for both INJURY and rGO groups. Scale bars: 50 μm. **(C)** Quantitative data of cavities present at the lesion site. Arrows set indicating the orientation of the tissue sections.

These positive electrophysiological recordings obtained from these 4 neurons activated by caudal-to-lesion stimulation prove the capacity of these rGO scaffolds to behave as a permissive environment for the regrowth of functional axons through the lesion in complete transected rats. Electrical measurements of these scaffolds provided a conductivity of 470 ± 78 mS m^−1^ and a sheet resistance of 0.55 ± 0.1 kΩ/sq (Fig. S1), being morphological, chemical and mechanical features prior to implantation similar to those previously reported [12] (Fig. S1). To identify differential anatomical features that could justify the existence of these respondent neurons in 27.3 % of SCI + rGO rats but not in SCI animals, extensive histological examination of the spinal cord tissue of these same rats was next performed. Based on the segregation of rGO-implanted rats into respondent and non-respondent animals from electrophysiological recordings, quantitative measurements were grouped into control (healthy), SCI (injured animals without scaffolds), SCI + rGO_NO-RES_ (rGO-implanted animals without positive responses in electrophysiological tests) and SCI + rGO_RES_ (rGO-implanted animals with positive responses in electrophysiological tests). First, we studied the spinal tissue after Masson's trichrome staining. A careful examination of the spinal cords proved the complete nature of the transection practiced in all injured animals (Fig. 3A). Larger magnification images revealed a significant abundance of collagen fibers populating both the interfaces and the lesion site in all damaged rats (Fig. 3B). Importantly, cavities could be identified in most of the lesions (∼30 % of the area of the spinal cord segment), and their extension was statistically similar among injured groups regardless of either the presence of the scaffold or the existence of positive electrophysiological features (one-way ANOVA, p = 0.84; Fig. 3C).

**Fig. 4:**
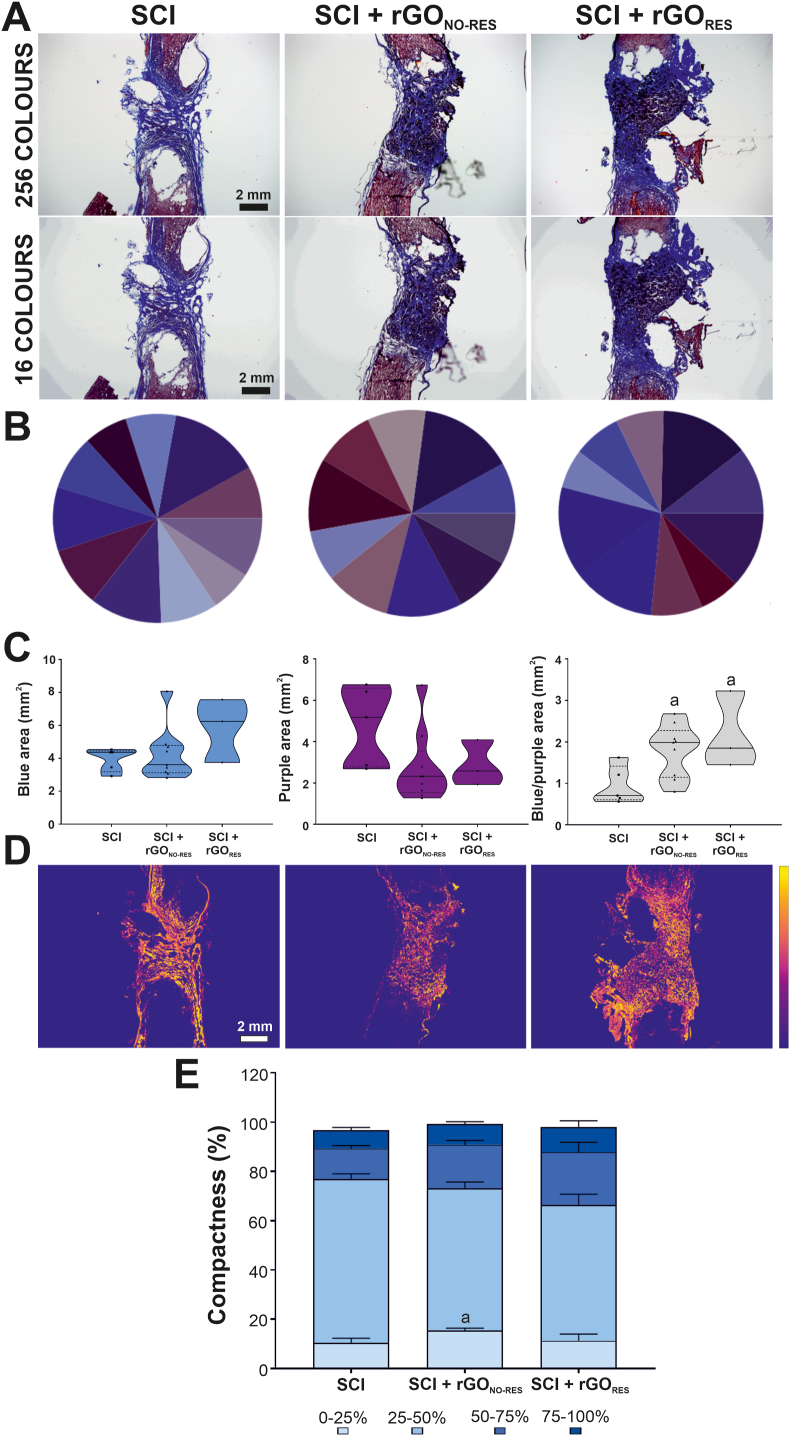
**Examination of collagen abundance and relative compactness in spinal cord tissue from transected rats receiving rGO scaffolds. (A)** Color transformation of Masson trichrome images (256 colours) into 16 colours-based ones. Scale bars: 2 mm. **(B)** Representative diagrams containing the different blue and purple tones identified in each group. **(C)** Quantitative data for the blue and purple areas and blue-to-purple ratios. **(D)** Representative transformed images illustrating the degree of collagen compactness in a thermal scale. **(E)** Quantitative data of collagen compactness. Statistics: one-way ANOVA followed by either Scheffé. Significance: p < 0.05 with respect to control (a).

The abundance of collagen fibers at the injured spinal cord required a more exhaustive characterization of this newly formed tissue. By using an automated protocol to avoid bias, we grouped the 256 original colours from each Masson's trichrome section into 16 colours (Fig. 4A). After excluding those assigned to background (image area without tissue), we calculated the relative presence of the remaining ones (typically, ⁓11–12 colours) with respect to collagen fibers (blue tones), preserved neural tissue (purple tones from hematoxylin staining), and rGO scaffold (black tones) (Fig. 4B and C). Results showed no statistically significant differences for blue and purple areas among injured groups. However, when expressed as a blue/purple ratio, injured animals carrying rGO scaffolds displayed significantly larger values than SCI rats regardless of their electrophysiological responsiveness (one-way ANOVA, p = 0.038∗). Although differences between respondent and non-respondent SCI + rGO rats were not significant, those animals with positive electrophysiological recordings showed a slightly higher ratio, corresponding to more collagen fibers in proportion to preserved neural tissue. Next, we measured the level of compactness from these blue-assigned pixels (collagen) (Fig. 4D and E). No differences were observed for any of the four levels of compactness established except for the smallest degree (0–25 %), which was significantly superior in SCI + rGO_NO-RES_ than in either SCI or SCI + rGO_RES_ rats (one-way ANOVA, p = 0.045∗). Based on this, the degree of compactness does not seem to correlate with the responsiveness of the rats carrying rGO scaffolds, although the abundance of collagen fibers could have an impact.

**Fig. 5:**
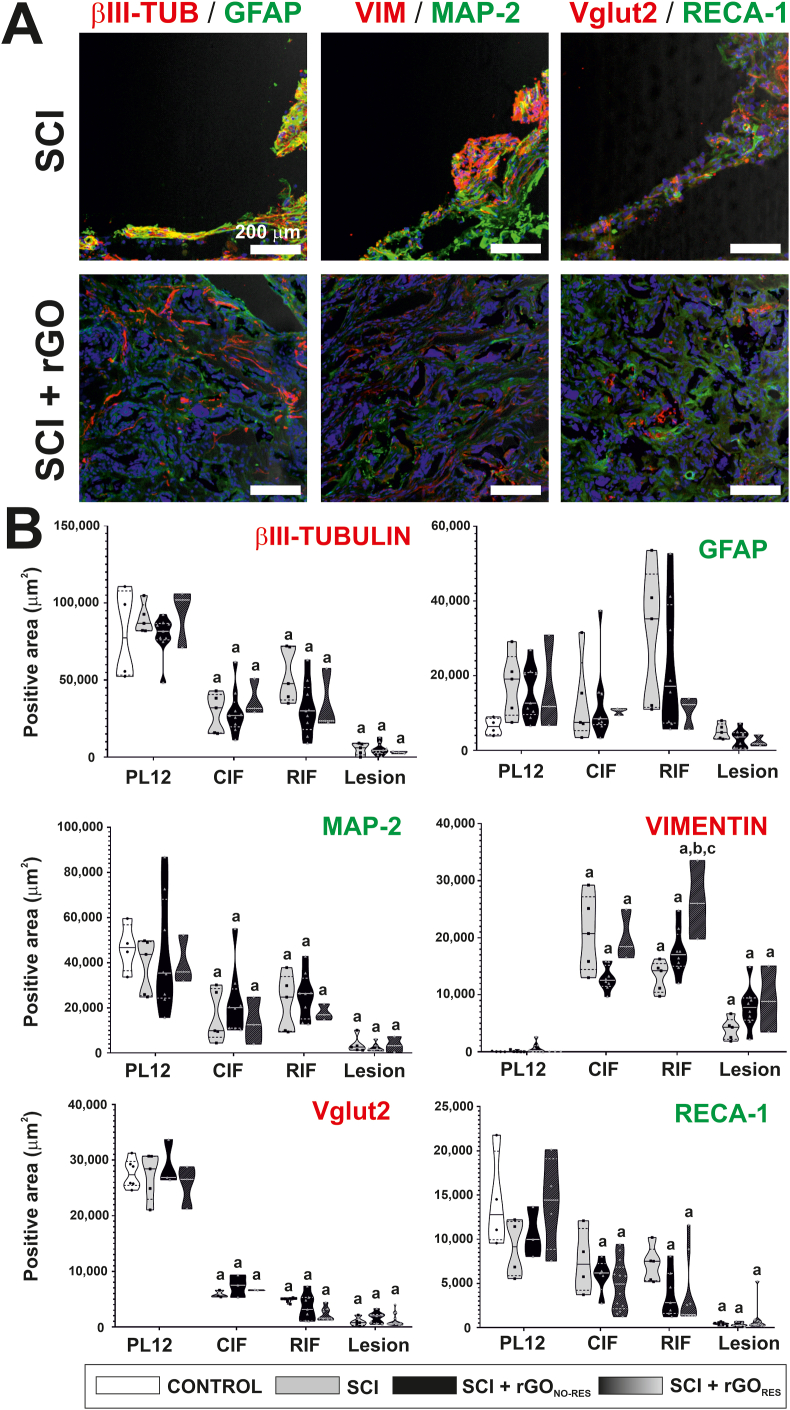
**Immunofluorescence characterization of the lesion site in paralyzed rats receiving rGO scaffolds. (A)** Representative confocal microscopy images at the lesion site for different markers under study as indicated. In all images, cell nuclei appeared in blue (Hoechst staining). Scale bar: 200 μm. **(B)** Respective quantitative data expressed as the positive stained area (%) from immunofluorescence images. Violin graphs were used in the representation of the data. Representative images for the remaining areas under investigation can be found in Figure S4, S5 and S6 βIII-TUB: βIII-tubulin; CIF: caudal interface; GFAP: glial fibrillary acidic protein; MAP-2: microtubule-associated protein 2; PL12: perilesional areas at 1–2 mm from the lesion border; RIF: rostral interface; VIM: vimentin. Statistics: one-way ANOVA followed by either Scheffé or Games-Howell *post hoc* tests (as dictated by Levene's test). Significance: p < 0.05 with respect to control (a), SCI (b) and SCI + rGO_NO-RES_ (c) groups.

**Fig. 6:**
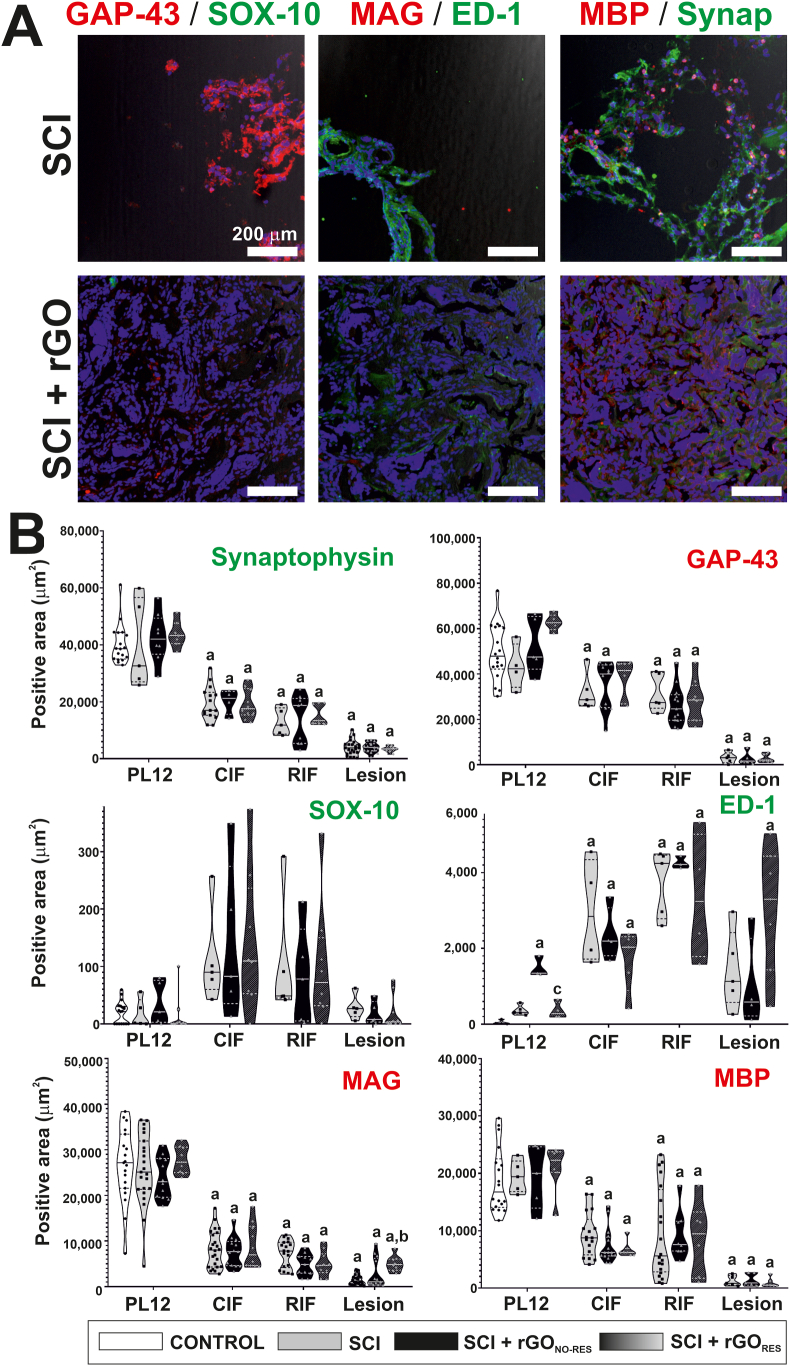
**Immunofluorescence characterization of the lesion site in paralyzed rats receiving rGO scaffolds. (A)** Representative confocal microscopy images at the lesion site for different markers under study as indicated. In all images, cell nuclei appeared in blue (Hoechst staining). Scale bar: 200 μm. **(B)** Respective quantitative data expressed as the positive stained area (%) from immunofluorescence images. Violin graphs were used in the representation of the data. Representative images for the remaining areas under investigation can be foundin Figure S4, S5 and S6. CIF: caudal interface; GAP-43: growth-associated protein 43; MAG: myelin-associated glycoprotein; MBP: myelin basic protein; PL12: perilesional areas at 1–2 mm from the lesion border; Synap: Synaptophysin; RIF: rostral interface; VIM: vimentin. Statistics: one-way ANOVA followed by either Scheffé or Games-Howell *post hoc* tests (as dictated by Levene's test). Significance: p < 0.05 with respect to control (a), SCI (b) and SCI + rGO_NO-RES_ (c) groups.

To further investigate the cell types populating the lesion site and its surroundings, we carried out an extensive immunofluorescence labelling with specific markers in four different anatomical areas (PL12, CIF, RIF, and lesion) (Fig. 5, Fig. 6, representative images at the lesion site (A) and respective quantification (B); representative images of the control in Fig. S3 and representative images of remaining anatomical areas for SCI and SCI + rGO groups in Figure S4, S5 and S6).First, neural components were labeled for βIII-tubulin and MAP-2 (Figs. 5 and S4), with not statistically significant differences within each specific area under study among groups (one-way ANOVA, βIII-tubulin: p = 0.67, p = 0.70, p = 0.11, and p = 0.66; MAP-2: p = 0.91, p = 0.71, p = 0.69, and p = 0.74; respective to PL12, CIF, RIF, and lesion). As expected, for both markers, there was a dramatic decrease in their abundance in all injured animals compared to control (uninjured) rats at the interface tissue and lesion epicenter (one-way ANOVA, p ≤ 0,001∗∗∗ for all groups), but not at PL12 (one-way ANOVA, p = 0.517 for βIII-tubulin and p = 0.918 for MAP-2). More importantly, neurites were present inside the lesion site in all injured animals, potentially enabling neural repair. In the case of the vesicular glutamate transporter VGLUT2 (Figs. 5 and S5), no significant differences were found for any comparisons among groups except for the expected reduction driven by the lesion (one-way ANOVA, p ≤ 0,001∗∗∗ for all groups in comparison to control rats). Astrocytes (labeled as GFAP^+^ cells; Figs. 5 and S4) were more abundant in all regions in injured rats than uninjured ones, regardless of the presence of the rGO scaffold, which did not significantly enhance their presence. Although not statistically significant due to the high dispersion of some of the groups, the area covered by GFAP^+^ cells showed a reduction trend in all CIF, RIF and lesion in rGO_RES_ animals with respect to both SCI and SCI + rGO_NO-RES_. Vimentin was also markedly enhanced by the injury, independently of the presence of the scaffold (one-way ANOVA, p ≤ 0.05∗ in all comparisons; Figs. 5 and S4). However, it showed an opposite trend in SCI + rGO_RES_ than GFAP, being the amount of vimentin at RIF statistically larger in these animals than injured ones without scaffolds (one-way ANOVA, p = 0.002∗∗∗) and SCI + rGO_NO-RES_ (p = 0.029∗). The area occupied by vascular structures (RECA-1^+^) was comparatively similar among groups at the four areas under investigation (Figs. 5 and S5), the areas affected by the lesion (CIF, RIF and lesion) markedly decreasing the abundance of vessels with respect to uninjured tissue (statistically significant for SCI + rGO_NO-RES_ and SCI + rGO_RES_, p ≤ 0.010∗; but not for SCI, p = 0.065 for CIF and p = 0.057 for RIF). Remarkably, blood vessels were present inside the lesion site in all injured animals, fundamental for nourishing neural tissue repair.

The presence of synapses, axon growth cones, Schwann cells, and myelin was also investigated (Fig. 6, representative images at the lesion site (A) and respective quantification (B); representative images of the control in Fig. S3 and representative images of remaining anatomical areas for SCI and SCI + rGO groups in Figs. S5 and S6). As expected, the abundance of synapses labeled by synaptophysin diminished at the lesion site and its proximities in comparison to healthy spinal tissue (one-way ANOVA, p ≤ 0,001∗∗∗ for all comparisons). Regarding growth axon cones (GAP-43^+^), similar findings were obtained except for CIF in SCI + rGO_RES_ rats, for which the decrease did not reach statistical significance with the control (p = 0.155). Excluding that, not significant differences were found among all injured groups at any of the areas of interest, neither for synaptophysin nor GAP-43. The presence of Schwann cells (SOX-10^+^) was almost negligible in all conditions, discarding any contribution of this cell type eventually coming from the sensitive dorsal roots into the reparative features observed. As expected, the abundance of proinflammatory macrophages (ED1^+^) was significantly enlarged in comparison to healthy spinal tissue at the interfaces (one-way ANOVA, p ≤ 0.05∗ in all comparisons). PL12 in all injured rats seemed to be slightly augmented compared to control rats, although only statistically significant for SCI + rGO_NO-RES_ rats (one-way ANOVA, p < 0.001∗∗∗). The increase in macrophages at the lesion site in SCI + rGO_RES_ showed a slight statistical significance with respect to control tissue (p = 0.012∗), but not for the other injured rats. Finally, myelin proteins MAG and MBP significantly diminished their abundance as a consequence of the lesion (one-way ANOVA, p < 0.001∗∗∗ for all comparisons with the control except for MBP in SCI + rGO_RES_ at RIF: p = 0.037∗). However, these markers did not show significant differences among injured rats in any region under study, with the exception of MAG at the lesion site in SCI + rGO_RES_ animals, which was augmented with respect to SCI rats (one-way ANOVA, p = 0.001∗∗∗), but not with SCI + rGO_NO-RES_ animals (one-way ANOVA, p = 0.129).

**Fig. 7:**
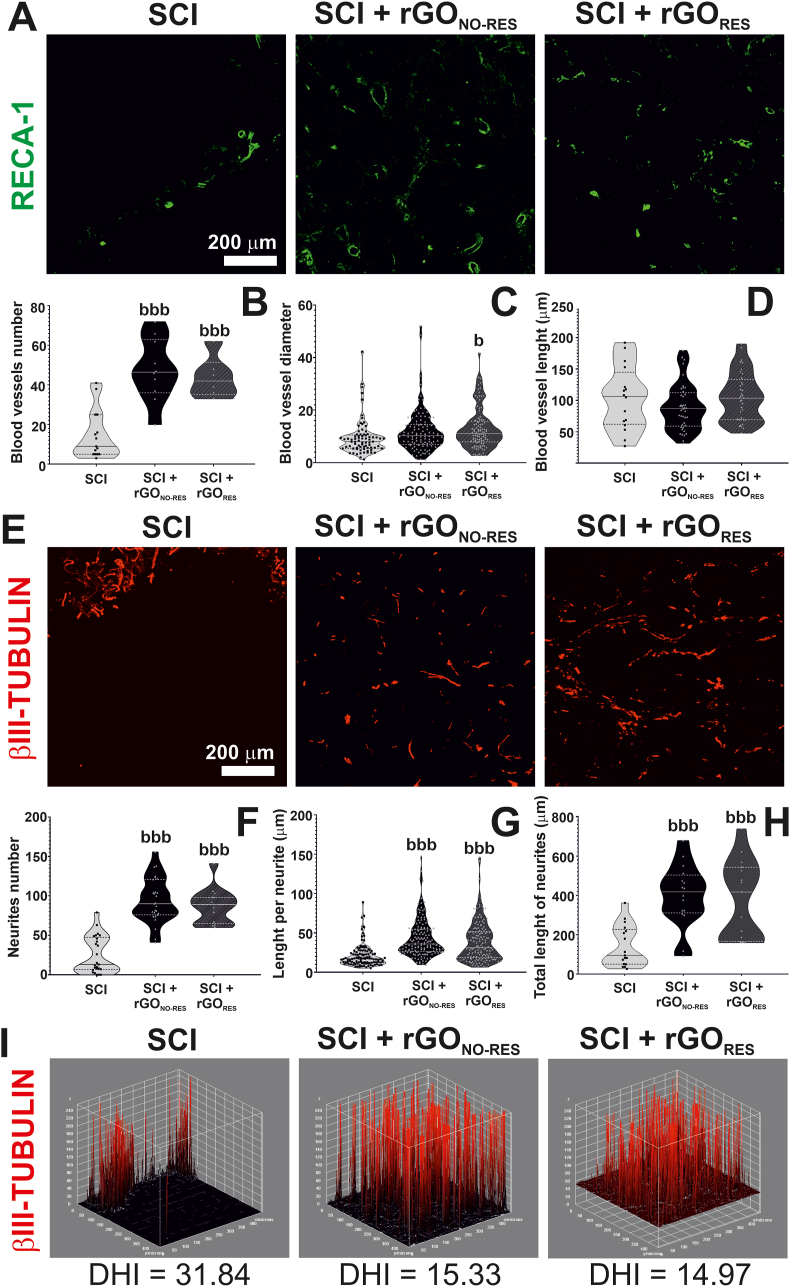
**Examination of blood vessels and neurites populating the lesion site in paralyzed rats receiving rGO scaffolds. (A)** Representative confocal microscopy images of blood vessels (RECA-1^+^). Scale bar: 200 μm. **(B**–**D)** Respective quantitative data expressed as the average of blood vessels number, diameter and length, respectively. **(E)** Representative confocal microscopy images of neurites (βIII-tubulin^+^). Scale bar: 200 μm. **(F**–**H)** Respective quantitative data expressed as the average of neurites number per image, length per neurite and total length per image, respectively. Violin graphs were used in the representation of the data. **(I)** Representative 3D reconstruction plots for images shown in (E). Based on electrophysiological results, the analysis of both blood vessels and neurites colonizing the rGO scaffold was disaggregated into respondent (SCI + rGO_RES_; X6, X21 and X23) and non-respondent (SCI + rGO_NO-RES_; all the rest) rats. Statistics: one-way ANOVA followed by either Scheffé or Games-Howell *post hoc* tests (as dictated by Levene's test). Significance: b < 0.05 and bbb <0.005 with respect to SCI.

As the twelve different markers investigated did not reveal a substantial difference among injured groups which could support their diverse responsiveness in electrophysiological recordings, we explored further two pivotal features at neural tissue repair: vascularization and neurite growth. First, RECA-1^+^ structures were measured in terms of number, diameter and length (Fig. 7A). Interestingly, the number of blood vessels significantly increased in all paralyzed rats receiving rGO (one-way ANOVA, p < 0.001 with respect to SCI), regardless of their responsiveness (one-way ANOVA, p = 0.85; Fig. 7B). On the contrary, the diameter of the blood vessels at the lesion site was statistically superior in SCI + rGO_RES_ animals (one-way ANOVA, p = 0.01 with respect to SCI), but not for SCI + rGO_NO-RES_ (one-way ANOVA, p = 0.10; Fig. 7C). The total length of blood vessels per image was similar among injured groups (one-way ANOVA, p = 0.17; Fig. 7D). Further quantification was next carried out for neurites invading the lesion site (Fig. 7E). Specifically, the number (Fig. 7F), the rostro-caudal length per neurite (Fig. 7G) and the total length of neurites per image (Fig. 7H) were all statistically higher in rGO-implanted rats regardless of their responsiveness in electrophysiological studies (one-way ANOVA, p < 0.001 in all comparisons with SCI). However, no substantial differences were found in these parameters between respondent and not-respondent rGO rats (one-way ANOVA, p = 0.67 for number, p = 0.27 for length and p = 0.95 for total length). Importantly, these neurites invading the rGO scaffolds were not only more abundant and longer, but also more homogeneously distributed within the lesion site in comparison to lesions without rGO as denoted by their respective distributional homogeneity indexes (DHI; control: 16.96 ± 2.67, SCI: 31.84 ± 10.11, SCI + rGO_NO-RES_: 15.33 ± 4.71, SCI + rGO_RES_: 14.97 ± 2.99; one-way ANOVA, p < 0.001∗∗∗ for SCI with all the groups) (Fig. 7I and Fig. S7). As for previous parameters, differences between respondent and not-respondent rGO-implanted rats were statistically negligible.

### 3.3 Behavioral assessment

**Fig. 8:**
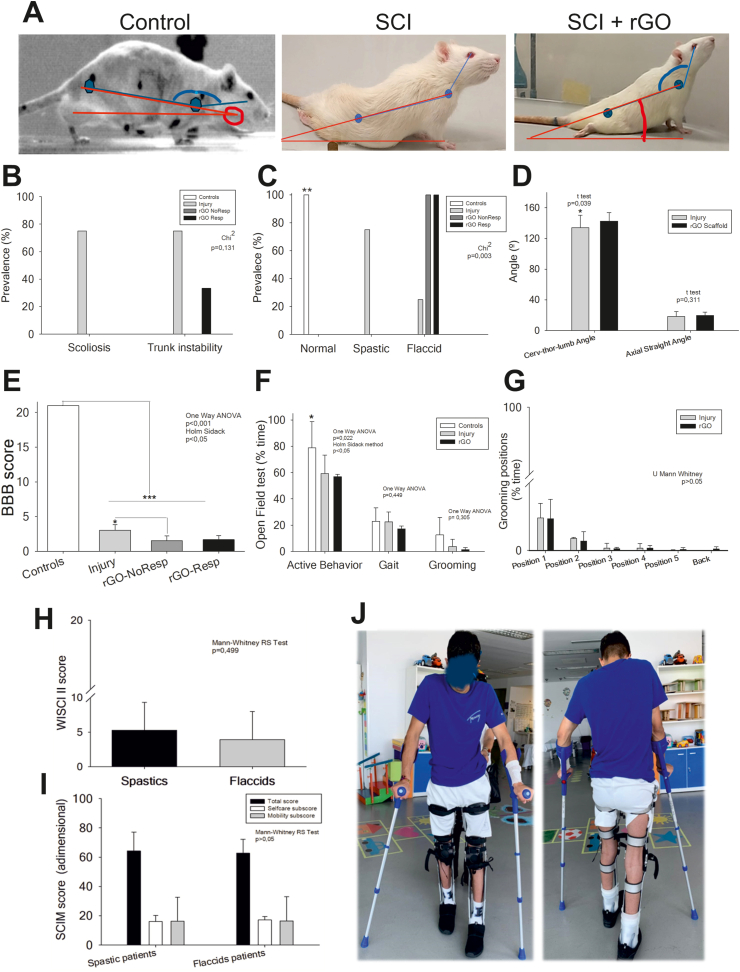
**Behavioral examination in transected rats after 4 months of rGO implantation and patients' series. (A)** Representative photographs of a control rat and transected rats with and without rGO scaffold implant. The body alignment variables defined have been represented on each of the three images: the *angle of trunk alignment* in red and the *cervico-thoraco-lumbar angle* in blue. **(B)** Prevalence of scoliosis and trunk instability in transected rats. Statistics: χ^2^, p = 0.131. **(C)** Prevalence of spastic and flaccid muscle tone in transected rats. Statistics: χ^2^, p = 0.03. **(D)** Quantitative analysis of the angular alignment measures in transected rats. All rGO-implanted rats were grouped together due to similar results. Statistics: *cervico-thoraco-lumbar angle*: T test, p = 0.039; *angle of trunk alignment*, T test, p = 0.311. **(E)** Quantitative analysis of locomotion behavior through BBB score in transected rats. Statistics: one-way ANOVA, p = 0.001; Holm-Sidak method, p < 0.05. **(F)** Quantitative analysis of Open Field Test in transected rats. Statistics: Active behavior: one-way ANOVA, p = 0.022; gait: one-way ANOVA, p = 0.449; grooming: one-way ANOVA, p = 0.305. **(G)** Quantitative analysis of the Grooming Test in transected rats. Statistics: Mann Whitney RS test, p > 0.05. **(H)** Quantitative analysis of the locomotion behavior of the patient's series through the WISCI II scale. Statistics: U Mann Whitney RS test, p = 0.499. **(I)** Quantitative analysis of the degree of functional independence in daily activities measured in the patients' cohort by using the SCIM III scale. Statistics: U Mann Whitney RS test, p > 0.05. **(J)** Representative photographs of a patient with complete thoracic paraplegia using orthoses and crutches.

Before carrying out the behavioral tests, all injured animals were inspected for the identification of spinal deformity (scoliosis, Fig. 8A) and assigned into three groups depending on the muscle tone of the hindpaws as follows: (1) normal, if the muscle tone of the hindpaws was similar to that of the forepaws; (2) spastic, if higher; and (3) flaccid, if lower. It is well established that the presence of spinal deformities often leads to trunk instability and poorer functional recovery [41], as the body axis plays a critical role in bipedal and quadrupedal locomotion, as well as during grooming in rats and daily living activities in humans [42]. The development of scoliosis was only evident among animals in the SCI group (Fig. 8B). As scoliosis leads to trunk instability in humans, we next defined *trunk instability* as the inability of the rats to release one of the forepaws in the position of maximum extension of the *cervico-thoraco-lumbar angle*. Trunk instability was largely found in SCI rats (*ca.* 80 %) and, to a less extent, in rGO_RES_ (*ca.* 30 %), but not in rGO_NO-RES_, although differences were not statistically significant (χ^2^, p = 0.131) (Fig. 8B). As expected, all thoracically transected rats, regardless of rGO scaffold implantation, had statistically significant and permanent changes in the muscle tone of their hindpaws compared to control animals (χ^2^, p = 0.003∗∗∗). Specifically, SCI rats were mostly spastic and SCI + rGO rats were only flaccid (Fig. 8C).

Thoracic spinal cord transection drastically altered whole-body alignment in the rats (Fig. 8A), as the hindpaws lost anti-gravity weight-support and displacement capacities. Although no differences in the *trunk alignment angle* were found among injured rats (T test, p = 0.311), a significantly lower *cervical-thoraco-lumbar* angular range of movement was confirmed in SCI rats in comparison to rGO animals (T test, p = 0.039∗; Fig. 8D). This finding corroborates a mechanical stabilization exerted by the rGO scaffold when implanted at the spinal cord lesion epicentre, in agreement with previous findings in cervical hemisected rats [12]. This positive effect clearly impacted motor skills of the forepaws and upper half of the trunk, which is the body region above the lesion able to develop significant motor compensations.

As expected, locomotor behavior was also severely altered as a consequence of the thoracic spinal cord transection. The BBB score was significantly lower in all injured rats with respect to control ones, independently of rGO scaffold implantation (one way ANOVA, p < 0.001∗∗∗; Fig. 8E). Noteworthy, the BBB score of the SCI group was significantly higher than that of rGO_NO-RES_ (p < 0.05∗), but not than that of rGO_RES_. Moreover, all injured animals showed a worsening in motor performance in the open field test, although only the decrease in active behavior was slightly significant compared to control rats (one way ANOVA, p = 0.022∗). No differences were either found between SCI and SCI + rGO groups in active behavior, gait and grooming (Kruskal-Wallis one way ANOVA on ranks, p = 0.05, p = 0.449 and p = 0.305, respectively; Fig. 8F). Finally, it is worth noting that only rGO rats were able to reach the back during grooming activities, the most biomechanically unfavorable position (Fig. 8G). Respondent and non-respondent rGO rats showed similar results in both open field and grooming behavior tests, without significant differences.

Proper evaluation and understanding of infralesional residual motor function in complete SCI subjects, being these either humans or animals, is usually challenging. The smaller the residual motor repertoire, the more difficult to assess its true significance. In order to interpret our experimental findings from a realistic clinical perspective, we next decided to check, in a human cohort, equivalent behavioral variables to those analyzed in the rats to elucidate if the results were similar, or at least comparable, and to understand their true translational significance. The epidemiological profile of the patients' series used in this study is summarized in Fig. S8, including demographic variables such as gender (Fig. S8A) and age and SCI time evolution (Fig. S8B) and the presence/absence of infralesional spasticity (Fig. S8C) and its score based on the Ashworth’ scale (Fig. S8D). All the selected patients, whether flaccid or spastic, suffered from complete paraplegia, with hardly any motor residual function in the lower limbs, as evidenced by their very low LEMS scores (Mann-Whitney Rank Sum test; p = 0.692) (Fig. S8E). The main differences found between the animal and human cohorts were: a) females were not included in the rat groups but present in the human cohorts; b) both rats and humans were adult individuals, but the humans were comparatively older; and c) all rats had a late chronic SCI (with the same time evolution), but the time evolution of human lesions, being the mean value also at the late chronic stage, was variable, with a chronicity range between 3 and 42 years.

Although the BBB scores revealed a significantly higher locomotor capacity in SCI and SCI + rGO_RES_ groups than rGO_NO-RES_, the clinical relevance of this result is unclear due to the overall low value reached in all groups. Moreover, these same two groups displayed infralesional spastic muscle tone. As the minimal change clinically relevant in the BBB score has yet to be defined, we decided to revise this data through a clinical human context, where the functional impact of the changes is easier to understand. Hence, we stratified the patients into spastic and flaccid and compared the residual locomotor capacity and the degree of functional independence for the activities of daily living. No statistically significant differences were found in either the WISCI II (Mann-Whitney Rank Sum test, p = 0.499; Fig. 8H) or the SCIM III (Mann-Whitney Rank Sum test, p > 0.05; Fig. 8I) scale scores between these two cohorts, neither in the total score (Mann-Whitney Rank Sum test, p = 0.403) nor in the selfcare (Mann-Whitney Rank Sum test, p = 0.967) and mobility subscales (Mann-Whitney Rank Sum test; p = 0.837; Fig. 8J). These findings in the human cohort question, at least partially, the clinical relevance of the gait differences found in rat behavior.

## 4 Discussion

Previous work by our group proved the ability of rGO scaffolds to promote neural repair features at the injured spinal cord in chronic cervical hemisected rats in a time dependent manner [11,12]. Herein, we further tested these matrices in a complete lesion, rather than an incomplete one, and a different spinal level (thoracic *vs.* cervical) at the time point with the larger quantity of reparative features found (*i.e.,* 4 months of implantation), including more abundant blood vessels, higher density of regrown neurites, and higher maturity in terms of myelination degree in regrown axons inside the scaffold, among others. In order to answer some relevant questions from previous studies, we included electrophysiological measurements to elucidate both the functionality of the axons regrowing inside the scaffolds and the origin of such neural fibers. Several brain structures such as cortex, cerebellum, midbrain, pons, and medulla, as well as descending propriospinal neurons, project axons to the spinal cord and are suitable for regeneration after a SCI [[43], [44], [45], [46]]. Our results showing antidromic activation of neurons located in brainstem nuclei in response to electrical stimuli applied caudally to the injured spinal cord constitutes a proof of concept that these rGO scaffolds support the ingrowth of axons that are physiologically active (*i.e.,* able to produce action potentials). Based on the stereotaxic coordinates of the recording neurons, the Gi of the reticular formation (RF) and the vestibular nuclei are the origin regions of these active projects. These results reinforce previous findings indicating RF as a primary structure involved in functional recovery after SCI [45,47], while extending the phenomenon to other structures is the brainstem as the vestibular nuclei, a region that plays a key role in balance and motor coordination [48,49]. Furthermore, recent work by Courtine and co-workers has demonstrated that Vsx2^+^Hoxa10^+^ spinal interneurons, a subtype of V2a interneurons that are located in the lumbar spinal cord, are crucial for the recovery of the locomotion after traumatic spinal cord injury in a mid-thoracic contusion [50]. These interneurons, which exclusively project to other interneurons in the ventral spinal cord, might have a pivotal role in the reparative features found in our case as they receive direct synaptic projections from parvalbumin-expressing neurons from the dorsal root ganglia and from neurons located in the ventral Gi nuclei. Additionally, Vsx2^+^Hoxa7^+^Zfhx3^+^ interneurons, located in the ventral mid-thoracic spinal cord and projecting longer distances to the lumbar spinal cord, also receive projections from the vGi [18]. Although related to the recovery of the behavioral outcome after incomplete SCIs, they might also have some kind of participation in our complete transection model.

Regarding neurons from the vestibular nuclei, our data indicates complex responses to rostral stimulation, involving multiple neurons with distinct properties (*i.e.,* conduction velocities). Importantly, the antidromic responses to rostral stimulation of neuron #3 and neuron #4 show unequivocal identification of neurons from vestibular nuclei. On the contrary, the caudal stimulation was not able to antidromically activate the axonal projections but generate a small local field potential corresponding to a synaptic activated neuronal cluster. This is observed in the two signals with constant latencies (7 and 14 ms), reduced efficiency (19 % and 41 %) and variable response magnitudes. Taking into account that the rostral stimulation showed an antidromic axonal activation, we consider that other axons from neurons in the same vestibular nuclei - that are different from the one recorded and activated by the rostral stimulation - could be invading the scaffold. These invading axons would then be activated by caudal stimulation, that in turn would generate a synaptic response in the recording neuronal population through axonal collaterals within the vestibular nucleus.

With this evidence of neural function, we next examined the spinal cord tissue to identify differential anatomical features supporting the recovery of neuronal functionality in rGO-implanted rats. After verifying that rGO did not modify the size of the cavities, we focused on collagen, a key protein of the extracellular matrix and markedly abundant at the lesion site in injured animals. Quantitative examination showed a significantly higher blue/purple ratio for SCI + rGO rats, indicating an increased amount of collagen at the injury site. Since collagen is known to play a role in neuronal regeneration by both providing biophysical stability to guide the direction of neuronal growth and promoting the adhesion and migration of neuronal cells [51], its presence at the injured area may facilitate the ingrowth of neurites in the scaffold, which was completely colonized by this protein. The investigation of neural, vascular and inflammatory markers at the spinal cord tissue did not reveal significant differences among experimental groups either. Only minor variances were observed and mostly located at the interfaces. For instance, respondent SCI + rGO animals showed slightly but a significantly larger amount of vimentin and a lower of VGLUT2 at RIF than injured animals without rGO implants. In these electrophysiologically positive animals, GAP-43 was found similar to control values at CIF, but not for injured or no respondent rGO animals. GFAP, although not statistically significant, showed a decrescent trend at both the rostral and caudal interfaces and the lesion site for respondent animals not found for the rest of injured rats. The abundance of proinflammatory macrophages in respondent rats slightly diminished at CIF, but significantly increased over the control at the lesion site. Myelin proteins were also homogeneously found among injured groups, except for a significant increase of MAG in SCI + rGO_RES_ with respect to SCI at the lesion site, not statistically different from rGO_NO-RES_. At the very lesion site, all injured animals seemed to possess similar histological features, none sufficiently diverse to support the electrophysiological recordings obtained.

We next focused on the examination of the two features that we identified as most likely related to this functional recovery: vascularization and neurite growth, both present at the lesion site in rGO-implanted rats. A careful analysis of the new blood vessels found inside the lesion site revealed statistically significant differences in terms of the number of blood vessels, being markedly superior for rGO-carrying animals. The length of those vessels was similar among groups, but their diameter was higher in respondent rGO rats with respect to injured rats without scaffolds. These results demonstrate a clear benefit of the implantation of rGO scaffolds at the injured spinal cord to permit angiogenesis and subsequent vascular remodeling, both essential phenomena for neural repair at the damaged central nervous system [52]. Since collagen was abundantly present at the lesion site, it is likely nourishing the growth of these new microvessels emerging from pre-existing ones. When considering neurites growth, outstanding differences were found among experimental groups. rGO-implanted animals displayed significantly more abundant and longer neurites at the lesion site. Moreover, they were more homogeneously distributed through the lesion (lower DHI), contrary to injured rats without implant, in which neurites mostly concentrated at the borders (higher DHI), in close contact with the interfaces. A similar staining distribution was obtained for all markers tested including vascular structures. Therefore, these findings reinforce the hypothesis that rGO implantation results in enhanced neural tissue repair through favoring the colonization of the scaffolds by regrown axons, thus contributing to the observed neuronal activation in our electrophysiological experiments.

All the histological measurements carried out reveal that rGO scaffolds promote anatomical features to help the ingrowth of neuronal projections in the transected spinal cord. These characteristics did not significantly differ in respondent and non-respondent rGO groups, indicating an overall pro-regenerative potential of this biomaterial. The fact that neurons with capacities to be antidromically activated were found in about a third of the rGO-implanted animals clearly shows an underestimation of the real numbers due to the complexity of the antidromic recordings and the use of tungsten electrodes for single-unit identification - instead of multielectrode array or shanks to cover a broader area and a higher neuronal population. Also, recordings were obtained only from brainstem. However, based on the high density of axon regrowth observed, it is highly probable that other descending tracts originated from different regions (*i.e.* sensorimotor cortex, the raphe nucleus, and propriospinal neurons, among others), and not targeted in this study, could be also invading the scaffold. Nonetheless, we herein report promising findings that show the nature of the invading axons promoted by the implantation of rGO scaffolds and the proof-of-concept that these neurons can be activated by an electrical current.

One could also think that the electrical current used for the recording in our study could be diffusing through the scaffold and triggering the activation of the rostral neurons. However, several lines of evidence confirm that the caudal stimulation was focal and activated axons within the scaffold and in the close proximity to the stimulation point without extending through the biomaterial. First, we observed a lower rate of neuronal activation during caudal stimulation of the injured spinal cord compared to rostral stimulation. This confirms that stimulation is local, with minimum spatial diffusion of the current. Second, we did not observe neuronal activation in response to caudal stimulation in injured animals without rGO scaffolds. Finally, in ideal physiological conditions, the electrical stimulation applied to different locations of an axon should result in the same efficiency of responses. However, we observed a reduced efficiency of antidromic responses from the caudal stimulation in comparison to the rostral one (*e.g.,* neurons #1 and #2). This reduction could be attributed to changes in the basic nature or structure of the axonal segments invading the scaffold such as the morphology of Ranvier nodes or the degree of myelination, which should be decreased inside the rGO scaffolds. Importantly, most of research work using biomaterials to promote axonal regrowth across a spinal cord injury use a massive electrical stimulation at the cranial level, which activates all descending tracts (including non-damaged ones in incomplete lesions) to record motor evoked potentials. Contrarily, in our work, we use the opposite direction of stimulation (*i.e.* antidromic), which allowed us to specifically and unequivocally identify the origin of axons able to regrow inside the rGO implanted scaffolds. This will facilitate to use the identified brain structures to selectively stimulate and promote axonal regrowth after SCI.

Besides mechanical compliance and biocompatibility previously described for these scaffolds [12], another important feature that could be assisting the creation of such permissive environment for repair at the injured neural tissue is an adequate conductivity degree. It is well known that the electrical conductivity of GBMs depends on pivotal chemical characteristics such as the C/O ratio and reduction degree [[53], [54], [55]]. Our rGO scaffolds, with a still large amount of oxygen-containing groups as denoted by their 0.28 % of C/O ratio (thermal annealing 200 °C for 30 min) [12], have a low conductivity of 0.47 ± 0.08 S m^−1^ and a sheet resistance of 0.55 ± 0.10 kΩ/sq. These values are more than two orders of magnitude below those described for 3D macroassemblies of graphene sheets as monolithic solids (*ca.* 100 S m^−1^) [56] and graphene sheets prepared by thermal exfoliation (7.3 % C/O ratio, 520 ± 360 kΩ/sq) [54]. Additionally, rGO powders with different thermal annealing conditions (400, 800 and 1000 °C) displayed conductivity values ranging from 117 ± 22 S m^−1^ (as received) to close to 1200 S m^−1^ (1000 °C of thermal annealing) [53], representing an up to 2000 times higher conductivity. However, from a regenerative point of view, one could wonder how useful this high electrical conductivity would be for an electrically active tissue such as the spinal cord, which is indeed injured at the time of the exposition to the conductive material. Indeed, too high conductivity values might not result beneficial but detrimental due to the potentiation of excitotoxicity in the remaining neuronal circuits which we intend to preserve and regenerate. Contrarily, electrically active materials with more biologically relevant conductivity values such as GBMs could be sufficient and more adequate to potentiate the functioning of neuronal circuits without harmful side effects, both at the central [10] and peripheral nervous system [57]. This still represents an open question in the race to provide an effective therapeutic solution not only for paraplegic patients but also for other patients suffering from severe injuries, either traumatic or non-traumatic, at the central nervous system and for which materials science and biomedical engineering could bring hope for a cure.

When integrated, the electrophysiological and histological findings demonstrating the invasion of the rGO scaffolds by axons from the reticular formation and the vestibulospinal tract are consistent with the significant improvement found in the axial straightening of the spinal cord rostral to the lesion, as measured by the *cervical-thoracic-lumbar angle*, also supported by the absence of scoliosis and a lower trunk instability. Differences within rGO-implanted animals, not explained either by electrophysiological, histological, or behavioral features, could be justified by a small but distinct longitudinal extent of the spinal cord damage, directly related to the degree of integrity of the circuits immediately above and below the lesion epicenter. Note that both axonal tracts identified in our results are originated in two brainstem nuclei involved in postural control as the Gigantocellular, in the reticular formation, and the vestibular nuclei. Although the postural improvement at the trunk level has been described as well for the corticospinal tract [58], the postural control directly depends on the brainstem descending reticulospinal and vestibulospinal axonal tracts. Therefore, the improvements in the posture control found in rGO animals is likely related to a better function of these pathways rostral to the scaffold mediated by its presence. In addition, diverse degrees of vascular compromise could be expected after the lesion. At this level, the Adamkievitz artery is the sole responsible for supplying the spinal anterior horn, including all the anterior and lateral descendent pathways and the motor neuron columns. Indirect injuries constitute the primary etiology of the ventral cord syndrome, being the resultant tissue damage secondary to the Adamkievitz ischemia [59]. Indeed, in human clinics, severe initial symptoms and a lack of improvement during the acute phase typically associates with worse recovery prognosis. Moreover, the larger the longitudinal vascular compromise within the spinal cord, the less potential for recovery. That is, the smaller the initial extent of the ischemia is, the more favorable functional prognosis [60]. As the anatomical integrity of the vasculature ventral to the spinal cord is not corroborated in our studies, we cannot neglect that differences found between rGO_NO-RES_ and rGO_RES_ animals could be related to slightly different degrees of Adamkievitz ischemia, then deriving into a lower potential for recovery regardless of the scaffold.

Our behavioral data also highlight that these rGO scaffolds play an important mechanical stabilizing role that extends beyond the spinal cord area in which it is implanted. The most obvious positive effect is the absence of scoliosis in the rGO-implanted rats in comparison to the high prevalence found in the SCI group. Trunk stability was also benefited by the presence of the rGO implant. Some works have indicated that neurons located two or three metameras rostral to the lesion site experience a general increase in their mechanical responsiveness, interpreted as a pathogenic factor associated with several clinical signs and symptoms such as pain [61]. In our model, this phenomenon of perilesional neuronal hypersensitivity could be modulated by the presence of the rGO scaffold, being responsible, at least partially, for the improvement in the axial straightening capacity of the implanted animals. Careful analyses of a cohort of human patients with comparable complete thoracic injuries did not provide substantial evidence that the prevalence of flaccid over spastic muscle tone in the hind paws of these complete transected rats could have a negative impact on the regenerative potential of these rGO scaffolds. Current work in our laboratory is focused on unravelling the molecular mechanisms behind the physico-chemical actuation provided by these 3D rGO scaffolds in the injured spinal cord, including genomic and proteomic studies. We hypothesize that, at least, some of the pro-regenerative routes that are enhanced by these biomaterials are related to mechanotransduction signaling pathways and, eventually, to their passive electrical properties.

## 5 Conclusions

Together, our results demonstrated the ability of rGO scaffolds to promote reparative features including the regrowth of blood vessels (more abundant and larger) and functional axons (more abundant, longer, more homogeneously distributed, and electrophysiologically active) in rats with a chronic thoracic transection. Antidromic electrophysiological recordings proved that some of these regrown neurites originated in the Gigantocellular nucleus of the reticular formation and the vestibular nuclei. Behavioral tests evidenced that these rGO scaffolds also play an important mechanical stabilizing role that extends beyond the spinal cord area in which they are implanted, as proved by the absence of scoliosis, a higher trunk stability and a larger cervico-thoraco-lumbar movement range in rGO-implanted rats. These findings corroborate the large neuro-reparative potential of these porous rGO scaffolds and strengthen the interest of their exploration for enabling repair at a highly specialized and electrically active tissue as the central nervous system.

## CRediT authorship contribution statement

**Marta Zaforas:** Writing – review & editing, Writing – original draft, Methodology, Investigation, Formal analysis. **Esther Benayas:** Writing – review & editing, Writing – original draft, Methodology, Investigation, Formal analysis, Data curation. **Raquel Madroñero-Mariscal:** Writing – review & editing, Methodology, Investigation, Formal analysis. **Ana Domínguez-Bajo:** Writing – review & editing, Methodology, Investigation, Formal analysis. **Elena Fernández-López:** Writing – review & editing, Methodology, Investigation. **Yasmina Hernández-Martín:** Writing – review & editing, Methodology, Investigation. **Ankor González-Mayorga:** Writing – review & editing, Methodology, Investigation. **Elena Alonso-Calviño:** Writing – review & editing, Methodology, Investigation. **Eduardo R. Hernández:** Writing – review & editing, Resources, Methodology, Investigation, Funding acquisition, Formal analysis. **Elisa López-Dolado:** Writing – review & editing, Writing – original draft, Supervision, Methodology, Investigation, Formal analysis, Data curation, Conceptualization. **Juliana M. Rosa:** Writing – review & editing, Data curation. **Juan Aguilar:** Writing – review & editing, Supervision, Resources, Methodology, Investigation, Funding acquisition, Formal analysis, Data curation, Conceptualization. **María C. Serrano:** Writing – review & editing, Writing – original draft, Supervision, Resources, Project administration, Methodology, Investigation, Funding acquisition, Formal analysis, Data curation, Conceptualization.

## Data availability

All data are available in the main text or the supplementary material. Additional raw and processed data required to reproduce these findings will be available to download from DIGITAL.CSIC upon acceptance and may also be requested from the authors.

## Ethics approval and consent to participate

Experiments were performed in accordance with the European Union guidelines (Directive 2010/63/EU). All processes were approved by the Ethical Committee for Animal Research at the *Hospital Nacional de Parapléjicos* and the *Dirección General de Agricultura y Ganadería* of *Castilla-La Mancha* (reference numbers 21–2016, 20–2021, and 3–2023).

## Funding Sources

This work has received funding from the 10.13039/100018693European Union's Horizon Europe research and Innovation Programme under grant agreement No. 101098597 (Piezo4Spine). It has been also supported by grant PID2020-113480RB-I00 funded by 10.13039/501100004837MCIN/10.13039/501100011033AEI/10.13039/501100011033/. The work carried out by ERH is supported by the Spanish State Funding Agency through project PID2022-139776NB-C66.

## Declaration of competing interest

The authors declare the following financial interests/personal relationships which may be considered as potential competing interests: Maria Concepcion Serrano reports financial support was provided by 10.13039/100018693Horizon Europe (PathFinder, 101098597, Piezo4Spine). Maria Concepcion Serrano reports financial support was provided by Spain 10.13039/501100004837Ministry of Science and Innovation (PID2020-113480RB-I00). Eduardo R. Hernandez reports financial support was provided by Spain 10.13039/501100004837Ministry of Science and Innovation (PID2022-139776NB-C66). Maria Concepcion Serrano is an associate editor for Bioactive Materials and was not involved in the editorial review or the decision to publish this article. Other authors declare that they have no known competing financial interests or personal relationships that could have appeared to influence the work reported in this paper.
