# Supplementary material for: Graphene oxide scaffolds promote functional improvements mediated by scaffold-invading axons in thoracic transected rats
Source: Bioact Mater. 2025 Jan 10;47:32–50. doi: 10.1016/j.bioactmat.2024.12.031 (PMC11772149; doi:10.1016/j.bioactmat.2024.12.031)
Supplement: Multimedia component 1 [file mmc1.docx]

**Supporting Information**

**
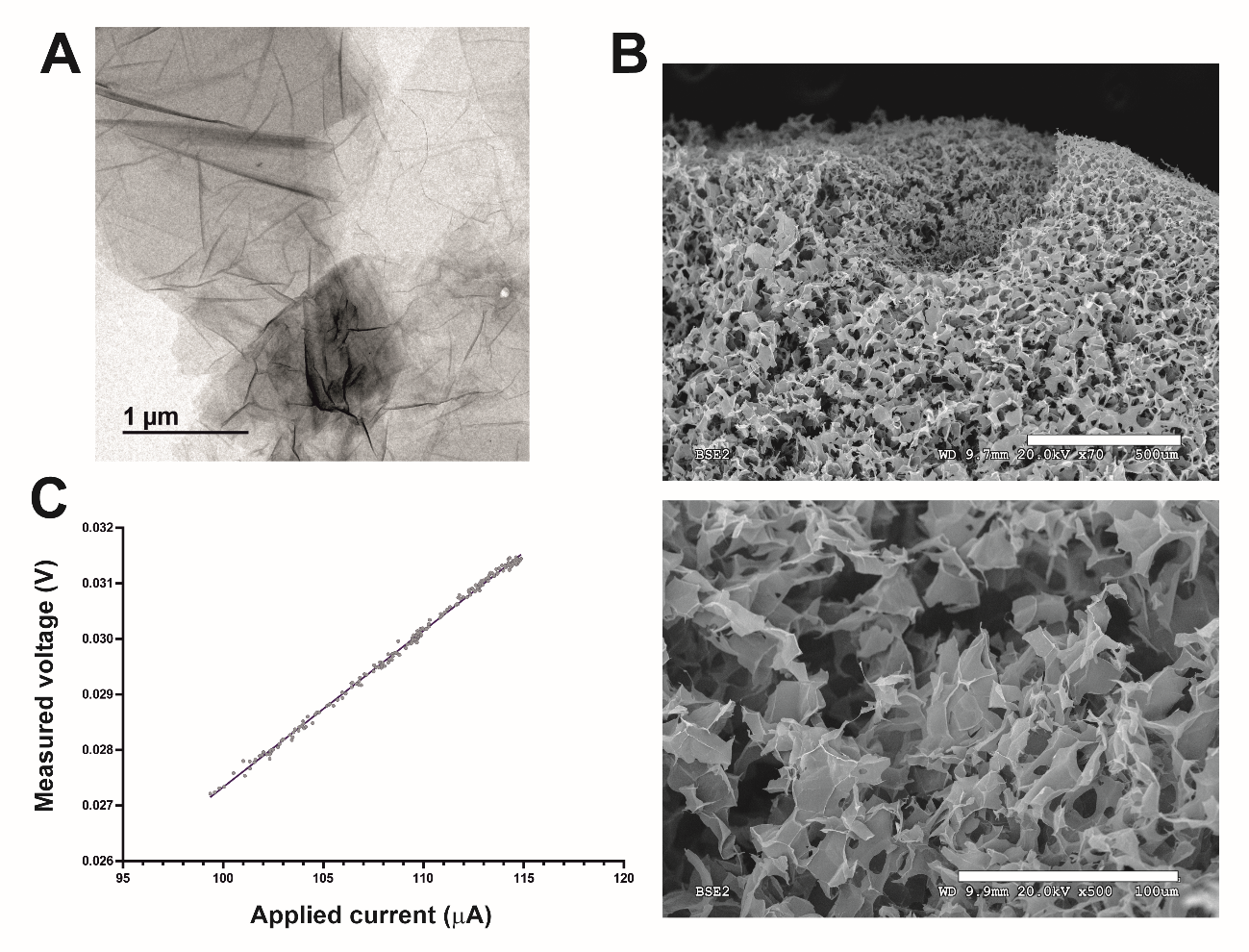
**

**Figure S1.** Physic-chemical characterization of rGO scaffolds. (a) Representative transmission electron microscopy image of some GO sheets in the commercial slurry used for scaffold fabrication. Scale bar: 1 μm. (b) Morphological characterization of resulting 3D rGO scaffolds by scanning electron microscopy. Scale bars: 500 μm (top) and 100 μm (bottom). (c) Voltage versus current curve obtained for rGO 3D porous scaffolds measured by four-point probe method. Each point represents a single measurement.


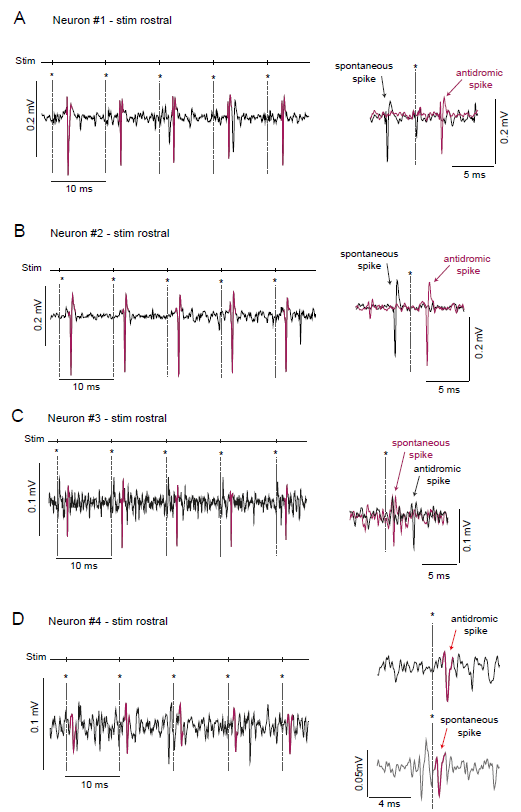


**Figure S2**. Neurons #1 to #4 were antidromically activated by rostral activation. Left, each neuron (from A to D, respectively), followed high frequencies (5 trials at 100 Hz) with antidromic responses shown in purple. Right, all four neurons passed the collision test: when a spontaneous orthodromic action potential occurred, antidromic activation was blocked.

**
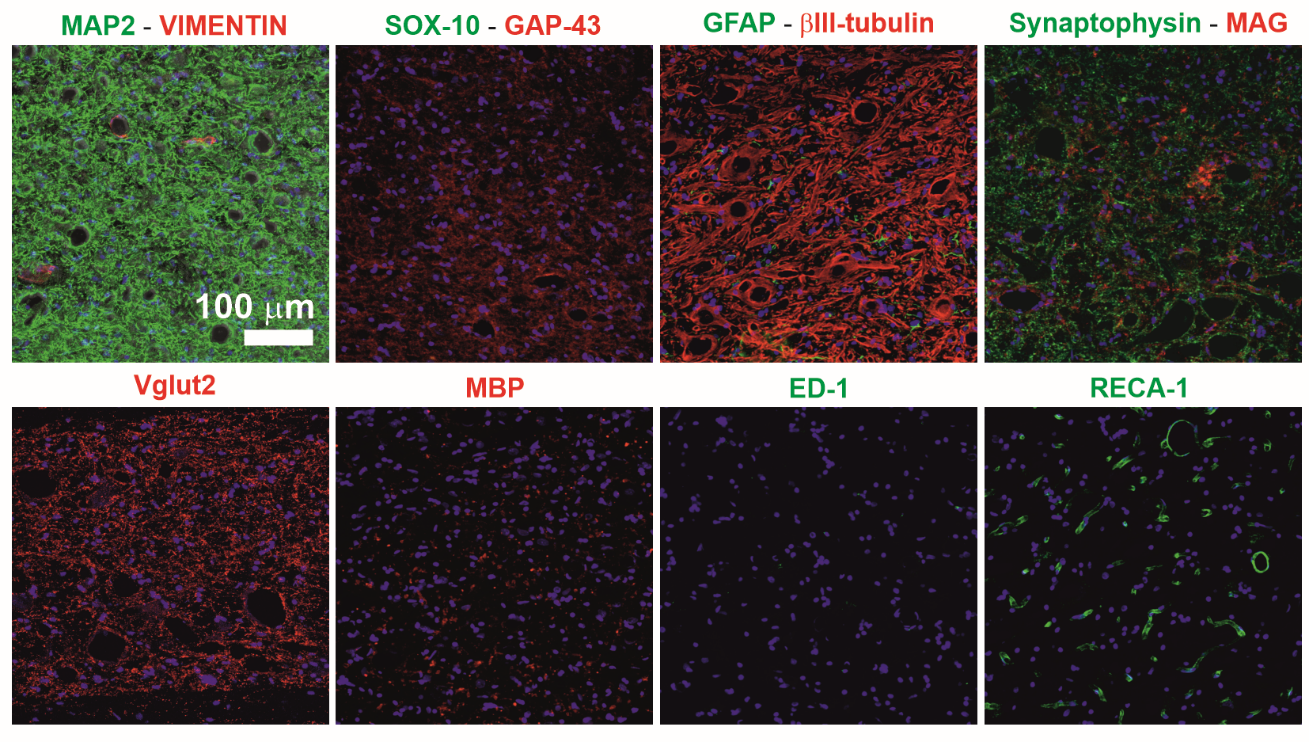
**

**Figure S3.** Immunofluorescence characterization of the grey matter of C6 spinal cord tissue of control (healthy) rats. Representative confocal microscopy images for the different markers illustrated in Figure 6 and Figure 7 as indicated. Reflection images are all merged with respective color channels. Scale bar: 100 μm.

**
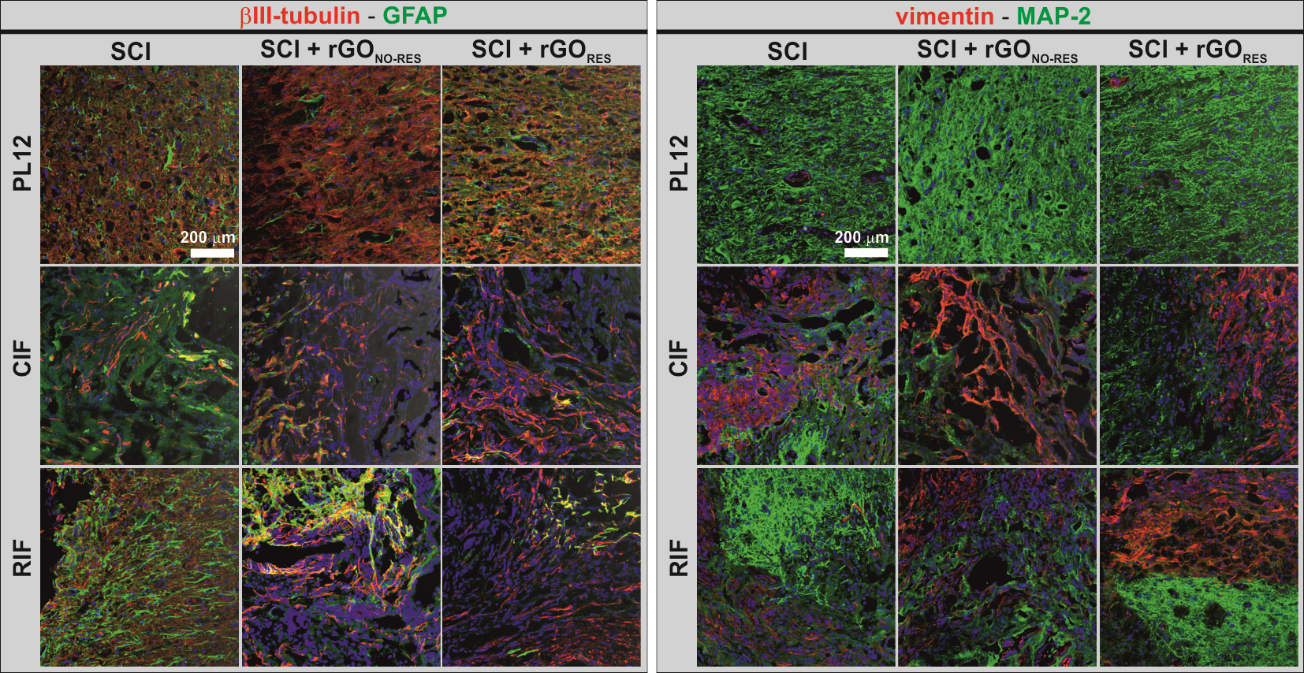
**

**Figure S4.** Immunofluorescence characterization of the spinal cord of paralyzed rats receiving or not rGO scaffolds in the different areas under investigation. Representative confocal microscopy images for βIII-tubulin, MAP-2, GFAP, and vimentin. Reflection images are all merged with respective color channels for scaffold visualization. Scale bar: 200 μm. CIF: caudal interface; PL12: perilesional areas at 1–2 mm from the lesion border; RIF: rostral interface.

**
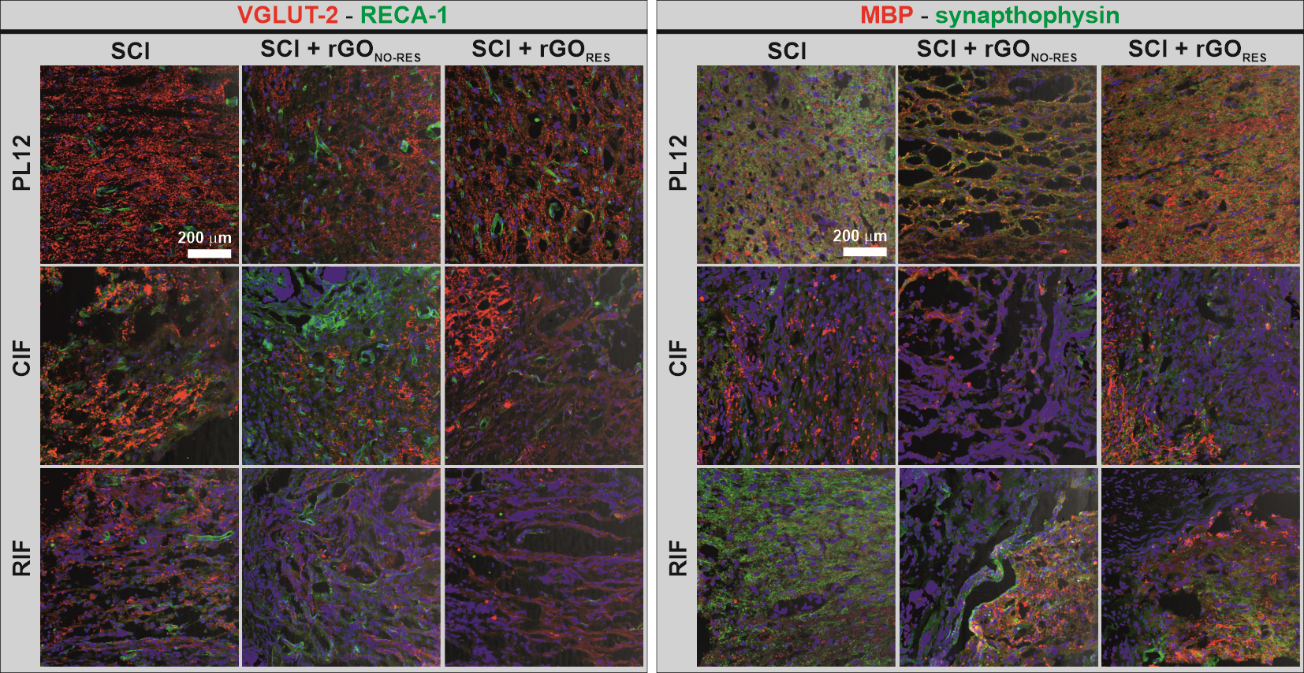
**

**Figure S5.** Immunofluorescence characterization of the spinal cord of paralyzed rats receiving or not rGO scaffolds in the different areas under investigation. Representative confocal microscopy images for synaptophysin, MBP, VGLUT-2, and RECA-1. Reflection images are also merged with respective color channels for scaffold visualization. Scale bar: 200 μm. CIF: caudal interface; PL12: perilesional areas at 1–2 mm from the lesion border; RIF: rostral interface.

**
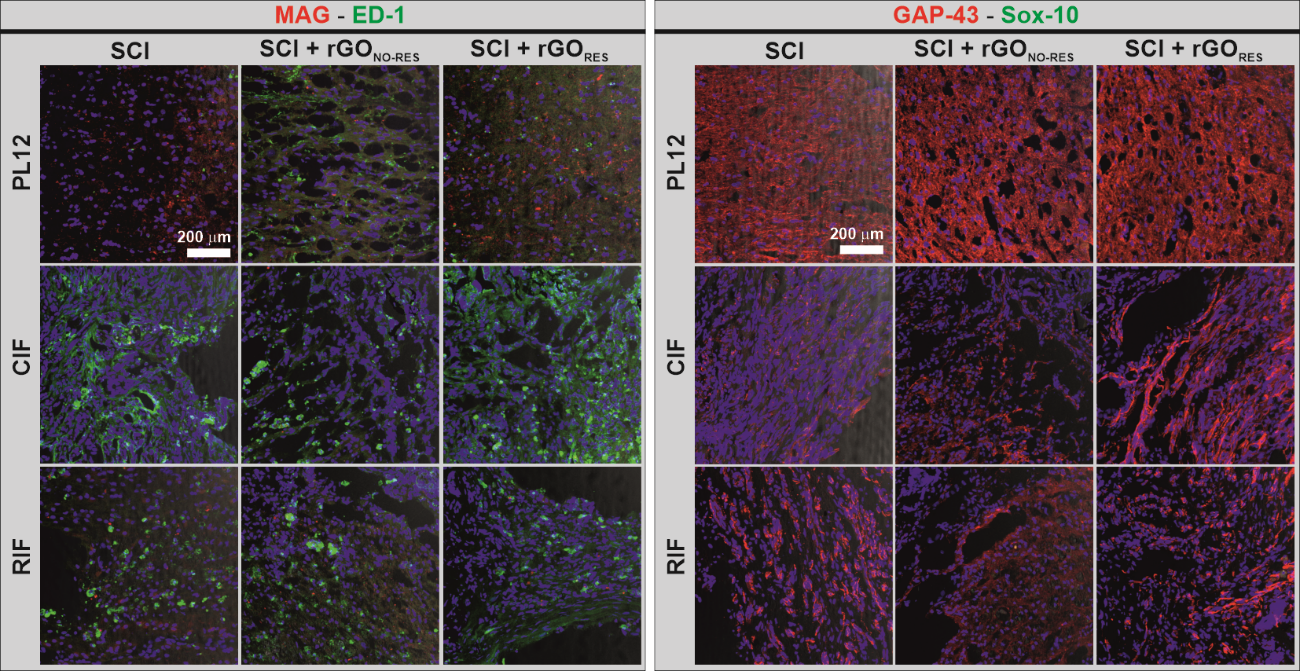
**

**Figure S6.** Immunofluorescence characterization of the spinal cord of paralyzed rats receiving or not rGO scaffolds in the different areas under investigation. Representative confocal microscopy images for MAG, ED-1, GAP-43, and Sox-10. Reflection images are also merged with respective color channels for scaffold visualization. Scale bar: 200 μm. CIF: caudal interface; PL12: perilesional areas at 1–2 mm from the lesion border; RIF: rostral interface.

**
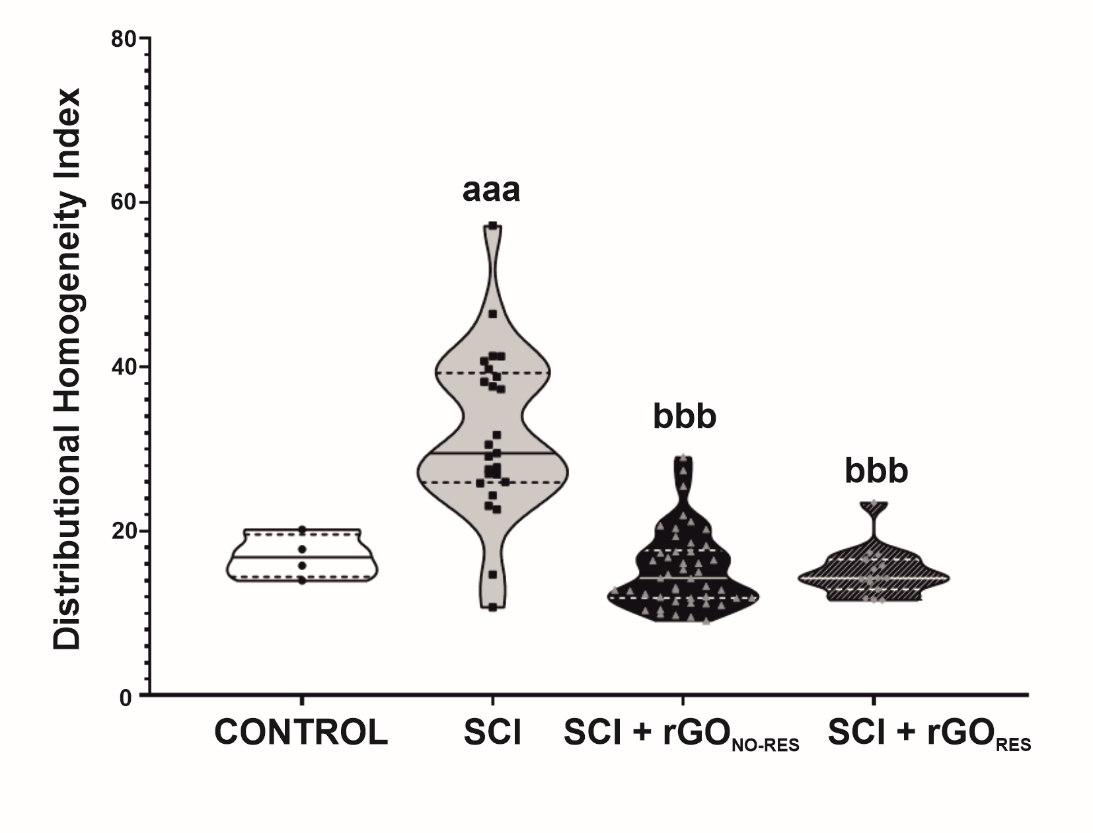
**

**Figure S7.** Distributional homogeneity index for βIII-tubulin at the lesion site in the different experimental groups. Statistics: one-way ANOVA followed by either Scheffé or Games-Howell post hoc tests (as dictated by Levene’s test). Significance: p < 0.005*** with respect to control (a) or injured (b) rats.

**
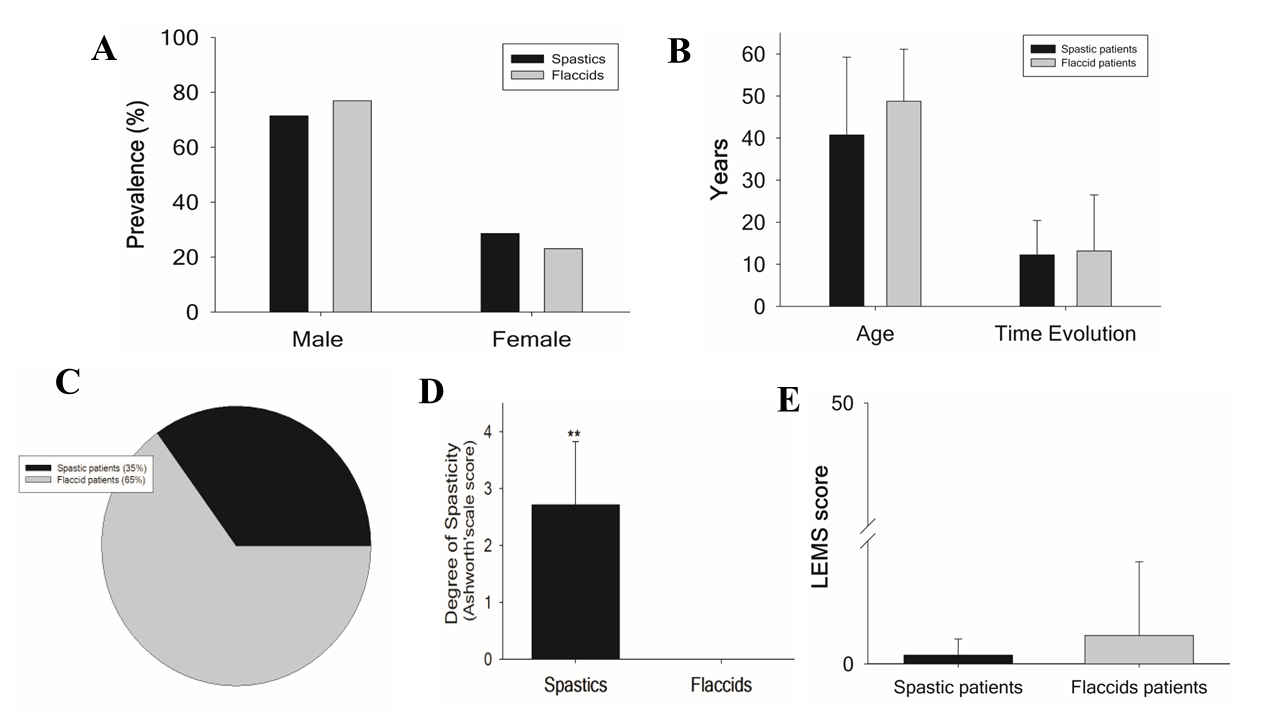
**

**Figure S8.** Quantitative analysis of some demographic and clinical variables of the patients’ cohort used in this study. **(A)** Distribution by gender among spastic and flaccid patients in the series. Statistics: χ^2^, p =1.000. **(B)** Age and SCI time evolution at recruiting. Statistics: Age: T test, p = 0.267; SCI time evolution: Mann Whitney RS test, p = 0.606. **(C)** Prevalence of spastics and flaccid patients in the series. Statistics: Mann Whitney RS test, p = 0.606. **(D)** Severity of the spasticity measured through the Modified Ashworth’ scale. Statistics: Mann Whitney RS test, p < 0.001. **(E)** Quantification of the zone of motor partial preservation through use of the LEMS score. Statistics: Mann Whitney RS test, p = 0.692.
